# Outcomes misaligned in mitochondrial encephalomyopathy, lactic acidosis and stroke-like episodes (MELAS): implications for trial design

**DOI:** 10.1093/braincomms/fcaf342

**Published:** 2025-09-09

**Authors:** Renae J Stefanetti, Sarah J Charman, Jane Newman, Kate Hallsworth, Alasdair P Blain, Yi Shiau Ng, Gráinne S Gorman

**Affiliations:** Translational and Clinical Research Institute, Faculty of Medical Sciences, Newcastle University, Newcastle upon Tyne NE2 4HH, UK; National Institute for Health and Care Research (NIHR) Newcastle Biomedical Research Centre (BRC), Newcastle upon Tyne NE4 5PL, UK; Newcastle upon Tyne Hospitals NHS Foundation Trust, Newcastle upon Tyne NE1 4LP, UK; Translational and Clinical Research Institute, Faculty of Medical Sciences, Newcastle University, Newcastle upon Tyne NE2 4HH, UK; Newcastle upon Tyne Hospitals NHS Foundation Trust, Newcastle upon Tyne NE1 4LP, UK; Translational and Clinical Research Institute, Faculty of Medical Sciences, Newcastle University, Newcastle upon Tyne NE2 4HH, UK; National Institute for Health and Care Research (NIHR) Newcastle Biomedical Research Centre (BRC), Newcastle upon Tyne NE4 5PL, UK; Newcastle upon Tyne Hospitals NHS Foundation Trust, Newcastle upon Tyne NE1 4LP, UK; NHS Highly Specialised Service for Rare Mitochondrial Disorders of Adults and Children, Newcastle upon Tyne Hospitals NHS Foundation Trust, Newcastle upon Tyne NE2 4HH, UK; Translational and Clinical Research Institute, Faculty of Medical Sciences, Newcastle University, Newcastle upon Tyne NE2 4HH, UK; National Institute for Health and Care Research (NIHR) Newcastle Biomedical Research Centre (BRC), Newcastle upon Tyne NE4 5PL, UK; Newcastle upon Tyne Hospitals NHS Foundation Trust, Newcastle upon Tyne NE1 4LP, UK; Translational and Clinical Research Institute, Faculty of Medical Sciences, Newcastle University, Newcastle upon Tyne NE2 4HH, UK; Translational and Clinical Research Institute, Faculty of Medical Sciences, Newcastle University, Newcastle upon Tyne NE2 4HH, UK; National Institute for Health and Care Research (NIHR) Newcastle Biomedical Research Centre (BRC), Newcastle upon Tyne NE4 5PL, UK; NHS Highly Specialised Service for Rare Mitochondrial Disorders of Adults and Children, Newcastle upon Tyne Hospitals NHS Foundation Trust, Newcastle upon Tyne NE2 4HH, UK; Directorate of Neurosciences, Royal Victoria Infirmary, Newcastle upon Tyne Hospitals NHS Foundation Trust, Newcastle upon Tyne NE1 4LP, UK; Translational and Clinical Research Institute, Faculty of Medical Sciences, Newcastle University, Newcastle upon Tyne NE2 4HH, UK; National Institute for Health and Care Research (NIHR) Newcastle Biomedical Research Centre (BRC), Newcastle upon Tyne NE4 5PL, UK; NHS Highly Specialised Service for Rare Mitochondrial Disorders of Adults and Children, Newcastle upon Tyne Hospitals NHS Foundation Trust, Newcastle upon Tyne NE2 4HH, UK; Directorate of Neurosciences, Royal Victoria Infirmary, Newcastle upon Tyne Hospitals NHS Foundation Trust, Newcastle upon Tyne NE1 4LP, UK

**Keywords:** mitochondrial, MELAS syndrome, stroke-like episodes, m.3243A>G, outcome measures

## Abstract

The m.3243A>G variant in the *MT-TL1* gene is the most prevalent pathogenic variant in mitochondrial DNA in adults, associated with a wide clinical spectrum from asymptomatic individuals to mitochondrial encephalomyopathy, lactic acidosis and stroke-like episodes syndrome. Although pharmacological trials in mitochondrial disorders are increasing, the lack of validated endpoints remains a significant barrier to therapeutic development. This cross-sectional observational study aimed to evaluate patients with and without mitochondrial encephalomyopathy, lactic acidosis and stroke-like episodes syndrome to identify factors associated with disease burden. Seventeen individuals genetically confirmed to harbour the heteroplasmic m.3243A>G pathogenic variant were enrolled: six who met the consensus-based diagnostic criteria for mitochondrial encephalomyopathy, lactic acidosis and stroke-like episodes syndrome (median age: 30.0 (inter-quartile range: 29.3–45.0) years). Ten patients who did not have a previous history of stroke-like episodes were assigned as ‘non-mitochondrial encephalomyopathy, lactic acidosis and stroke-like episodes’ (age: 37.5 (32.8–48.3) years). Of these patients in the non-mitochondrial encephalomyopathy, lactic acidosis and stroke-like episodes group, seven exhibited variable features of mitochondrial disease, including hearing loss, diabetes mellitus, migraine and gastrointestinal involvement, while the remaining three were asymptomatic. One patient was excluded from analysis due to a confirmed ischaemic stroke unrelated to mitochondrial disease. Assessments included disease severity (Newcastle mitochondrial disease adult scale) and patient-reported outcomes of fatigue (fatigue impact scale), health-related quality of life (Newcastle Mitochondrial-QoL), mental well-being (Warwick–Edinburgh mental wellbeing scale), autonomic symptoms (the composite autonomic symptom) and physical activity (The International Physical Activity Questionnaire). Performance outcomes included timed-up and go, handgrip strength, cardiopulmonary exercise testing and accelerometry. Age- and sex-matched healthy controls were included for comparison of accelerometry data (age: 35.5 (28.8–50.5) years). Despite comparable age and mitochondrial DNA heteroplasmy, patients with mitochondrial encephalomyopathy, lactic acidosis and stroke-like episodes syndrome had significantly higher disease burden, reduced exercise capacity and lower levels of objectively measured physical activity compared to non-mitochondrial encephalomyopathy, lactic acidosis and stroke-like episodes and controls (*P* < 0.05–0.001). Patient-reported outcomes did not significantly differ between mitochondrial encephalomyopathy, lactic acidosis and stroke-like episodes syndrome/non-mitochondrial encephalomyopathy, lactic acidosis and stroke-like episodes. While non-mitochondrial encephalomyopathy, lactic acidosis and stroke-like episodes patients showed expected alignment between perceived and objective measures, mitochondrial encephalomyopathy, lactic acidosis and stroke-like episodes syndrome patients demonstrated weak, absent, or paradoxical associations. This mismatch may reflect altered symptom perception, cognitive impairment, or disease-related adaptation. These findings underscore the complexity of disease expression in mitochondrial encephalomyopathy, lactic acidosis and stroke-like episodes syndrome. Regulatory agencies encourage the inclusion of patient-centred endpoints; however, this study highlights the potential limitations of relying solely on patient-reported outcomes. The divergence between subjective and objective assessments supports the need for multi-dimensional outcomes that integrate both patient perspectives and objective measures to enhance the reliability and interpretability of clinical trials in primary mitochondrial disease.

## Teaser

Stefanetti *et al*. studied adults with the m.3243A>G mitochondrial DNA variant and found that individuals with mitochondrial encephalomyopathy, lactic acidosis and stroke-like episodes (MELAS) syndrome had greater physical limitations, despite self-reporting symptoms similar to those without. This mismatch highlights the need for outcomes to capture both patient experience and objective function.

## Graphical Abstract

**Graphical Abstract:**
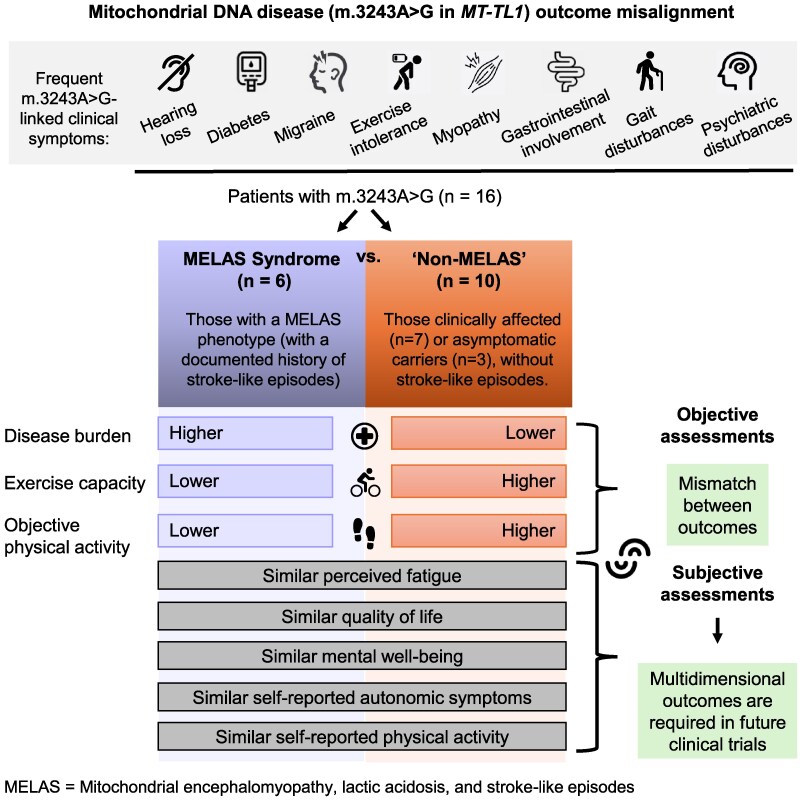

## Introduction

Primary mitochondrial diseases are a heterogeneous group of genetic disorders resulting from pathogenic variants in mitochondrial DNA (mtDNA) or nuclear DNA (nDNA) genes, essential for oxidative phosphorylation.^1^ Dysfunction in the mitochondrial respiratory chain impairs adenosine triphosphate production, affecting high-energy demanding organs, such as skeletal muscle, the heart and the nervous system.^2^

The pathogenic m.3243A>G mtDNA variant in the *MT-TL1* gene (encoding mitochondrial transfer RNA^leucine (UUR)^) is the most prevalent mtDNA-related disease genotype in adults.^3^ Its phenotypic spectrum ranges from severe multi-systemic conditions like Mitochondrial encephalomyopathy, lactic acidosis and stroke-like episodes (MELAS) syndrome, characterized by stroke-like episodes, to asymptomatic presentations.^2,4,5^ Ten percent^2^ to 45%^6^ of individuals harbouring the m.3243A>G pathogenic variant develop MELAS syndrome.^5^ Clinical variability is only partially understood. While mtDNA heteroplasmy offers some explanation,^7,8^ it does not fully account for the phenotypic spectrum frequently observed. Nuclear genetic background, environmental factors and epigenetic changes may also influence disease burden, highlighting the need for further research. With the increasing focus on clinical trials for mitochondrial disease^9^ (also see Supplementary A), clinically relevant and accurate outcome measures are pertinent for evaluating treatment efficacy.^10^

Fatigue and exercise intolerance are two of the most common and burdensome symptoms in m.3243A>G-related mitochondrial disease and are rated among the most important research priorities.^11-15^ Skeletal muscle involvement often results in low exercise tolerance and marked fatigue,^16^ limiting activities of daily living (ADLs) and leading to a sedentary lifestyle.^16,17^ This creates a cycle of physical inactivity and deconditioning, exacerbating symptoms and increasing comorbidities.^18^ Cardiopulmonary exercise testing (CPET) is the gold standard for evaluating exercise capacity,^19^ providing insights into the integrated physiological responses to exercise.^20^ However, its implementation is limited by the need for sophisticated equipment, settings and specialized staff.

In recent years, advances in technology have led to a shift towards increased virtual healthcare^21^ and remote/decentralized approaches to clinical trials.^22,23^ Wearable technologies, like wrist-worn accelerometers, offer continuous, real-world measurements of physical activity, inactivity and sleep outside traditional clinical settings,^24-27^ providing valuable insights into physical functioning and ultimately, disease progression.^28,29^

This study aims to identify factors influencing the m.3243A>G disease burden by investigating clinical, subjective and objective parameters, with a specific focus on distinguishing between MELAS syndrome and non-MELAS disease status. Given the devastating clinical trajectory and high disease burden associated with MELAS syndrome, this differentiation enabled exploration of phenotype-specific investigations between outcome measures. By examining correlates of disease severity, the study seeks to inform clinical trial design, delivery and drive meaningful improvements in patient outcomes.

## Materials and methods

### Participants

Seventeen adults (≥18 years, seven females) with the genetically confirmed m.3243A>G pathogenic mitochondrial DNA variant were recruited from the NHS Highly Specialised Service for Rare Mitochondrial Disorders in Newcastle upon Tyne, UK between September and December, 2016. Exclusion criteria included inability to undertake any of the assessments, as well as a history of vascular stroke unrelated to MELAS syndrome, brain lesions or tumours and significant but unrelated medical comorbidities, as determined by the physician at screening. One patient was excluded because, although they initially met the criteria, brain imaging revealed a vascular stroke, which did not meet the inclusion criteria for analysis. The full inclusion and exclusion criteria are provided in Supplementary B.

Six patients were categorized as MELAS syndrome three females, median age: 30.0 (inter-quartile range [IQR]: 29.3–45.0) years, median body mass index (BMI): 21.3 (IQR: 18.5–22.2 kg/m^2^), median Newcastle Mitochondrial Disease Adult Scale (NMDAS) score: 43 (IQR: 35.6–51.2) using the consensus-based definition of mitochondrial stroke-like episodes and diagnostic criteria based on clinical features (such as focal-onset seizures, encephalopathy, with or without additional focal neurological deficits) and corroborating neuroimaging and/or electroencephalogram findings.^5^ Ten patients with m.3243A>G who did not suffer from stroke-like episodes were designated as ‘non-MELAS’ (four females, median age: 37.5 (IQR: 32.8–48.3) years, median BMI: 24.3 (IQR: 22.8–28 kg/m^2^), median NMDAS score: 23.4 (IQR: 5.9–26.6). Of the 10 non-MELAS patients, seven were symptomatic and exhibited variable features of mitochondrial disease (NMDAS score: 25.9; IQR: 21.8–36.5) and three were asymptomatic carriers (NMDAS scores: 3.1, 4.1, 4.1, respectively).

Ethical approval was obtained from the Research Ethics Committee (REC) North East—Newcastle and North Tyneside 2 (REC REF: 15/NE/0399). Written informed consent was obtained from all participants prior to participation and all research assessments were conducted according to the principles expressed in the Declaration of Helsinki.^30^

### Study assessments

To comprehensively assess disease burden, multiple clinical outcome assessments were incorporated, including clinician-reported outcome (ClinRO), patient-reported outcomes (PROs) and performance outcomes (PerfOs) as defined by the U.S. Food and Drug Administration.^31^ ClinROs involve evaluations made by a trained clinician based on clinical observation or judgment. PROs capture the patient’s perspective on their health, functioning or symptoms, as reported directly by the patient without interpretation by clinicians or others. PerfOs are based on standardized tasks performed by the patient that are administered and evaluated by an appropriately trained individual.

### Clinician-reported outcomes

#### Demographics and clinical characteristics

Demographic and clinical information including sex, body weight, height, occupational status, smoking history, age at disease onset, stroke-like episode history and laboratory data (serum lactate) were collected. Diagnostic information, including m.3243A>G mtDNA heteroplasmy levels in blood and urine, was obtained from the patients’ medical records. mDNA heteroplasmy in blood and urinary sediment was quantified by pyrosequencing, as previously described and validated.^32^

#### Disease severity

Disease severity was assessed using the NMDAS, a semi-quantitative clinical rating scale designed to evaluate disease burden in all forms of adult mitochondrial disease.^33^ The rating scale encompasses all aspects of mitochondrial disease using the following domains: Current function, System Specific Involvement and Current Clinical Assessment including Wechsler Test of Adult Reading test, Symbol search and speed of comprehension test.

### Patient-reported outcomes

Patients completed paper-based questionnaires at the clinical research facility, independently where possible, with assistance from the study team provided when required.

#### Fatigue

The 40-item fatigue impact scale (FIS) assessed fatigue's impact on physical, cognitive and psychosocial functioning, with a maximum score of 160, where higher scores indicate greater fatigue impact.^34^

#### Health-related quality of life

The Newcastle Mitochondrial Quality of Life (NMQ) questionnaire, comprising 63 items across 16 life domains, measured mitochondrial disease-specific health-related quality of life (QoL), with scores ranging from 0 to 100, where higher scores reflect better QoL.^35^

#### Mental well-being

The 14-item Warwick–Edinburgh mental wellbeing scale (WEMWBS) assessed mental well-being, with total scores ranging from 14 to 70, where higher scores indicate better psychological functioning.^36^

#### Autonomic function

The composite autonomic symptom (COMPASS 31) was used to quantify autonomic symptoms across six domains, with weighted scores ranging from 0 to 100.^37^ Higher scores were indicative of higher autonomic dysfunction.

#### Physical activity

The International Physical Activity Questionnaire (IPAQ)—long form was used to assess self-reported physical activity over 7 days. Physical activity categories and total activity in MET (metabolic equivalent task)—minutes were summed according to the official IPAQ scoring protocol.^38^ Vigorous-intensity PA was assumed to correspond to eight METs, moderate-intensity activity to four METs and walking to 3.3 METs.

### Performance outcome measures

#### Functional capacity

The timed-up and go (TUG) test^39^ was performed as a measure of lower extremity functional mobility. The time to complete the task was measured and the average of three trials recorded with a faster time indicative of a greater functional performance. A Jamar® Hand Dynamometer was used to measure handgrip strength^40^ and grip strength recorded as the maximum force produced (in kilograms) of three attempts with their dominant hand.

#### Exercise capacity

A CPET was performed using analysis of expired air gases (Metalyzer® 3B-R3; Cortex, Leipzig, Germany) and non-invasive bioreactance cardiac output measurement (NICOM; Cheetah Medical, UK). Patients cycled on an electronically braked recumbent ergometer (Corival®; Lode, Netherlands) with a stepped incremental workload (∼10–20 W/min) to determine peak oxygen uptake and heart rate. Heart rate (12-lead ECG), respiratory gas exchange, Rate of Perceived Exertion (Borg 6–20) and blood pressure were monitored until volitional exhaustion. Peak exercise values were derived from the 30-second average at peak. The anaerobic threshold was assessed using the V-slope method,^41^ and arterial–venous oxygen difference (a-vO_2_ diff) was calculated from peak oxygen consumption and cardiac output as previously described.^42^

#### Accelerometry—physical activity, inactivity and sleep

Participants completed seven days of wrist-worn accelerometer monitoring (GENEActiv, ActivInsights Ltd, UK). Data were processed in R (www.cran.r-project.org) using the GGIR Rpackage (Version 2.7.0),^43-45^ with a minimum of 16 h wear time per day and at least three consecutive days of monitoring, including one weekend day.^46^ Raw acceleration signals were corrected for calibration error,^47^ and monitor non-wear periods^43^ were replaced with average data from similar time points on different days.^48,49^ The average magnitude of wrist acceleration per 5-second epoch was calculated using the metric ENMO (1 mg = 0.001×gravitational acceleration), as previously described.^43^ Activity was categorized as inactivity (<40 mg), light physical activity (40–100 mg) and moderate-vigorous physical activity (MVPA) (>100 mg).^50,51^ Time spent in 30-min inactivity bouts and 5–10 min MVPA bouts was calculated, along with daily step count and average sleep duration and efficiency, as described previously.^50-53^

A group of age- and sex-matched healthy controls (*n* = 16; seven females, age: 35.5 (28.8–50.5) years, BMI: 25 (21.5–26.6) kg/m²)) were used as a comparator, by performing accelerometry, as described above (Data obtained approved via Newcastle University Ethics Committee (2901/2017)).

### Statistical analysis

No sample size calculation was performed; the analyses were based on all available cases meeting the inclusion criteria. Data were analysed using SPSS (version 28, SPSS, Inc., Chicago, IL, USA). The level of significance was set at *P* < 0.05. Data are described as median (IQR) unless otherwise stated. Prior to statistical analysis, data were screened for normality and outliers using Shapiro–Wilks test and boxplots. After adjustment for BMI, Differences in acceleration categories between groups (MELAS syndrome, non-MELAS and healthy controls) were assessed using analysis of covariance (ANCOVA), adjusting for BMI, which was included as a covariate due to its known influence on physical activity levels and sleep characteristics. *Post-hoc* differences were identified using the Bonferroni test. For all other outcome measures, differences between m.3243A>G MELAS syndrome/non-MELAS patients were assessed using independent *t*-tests. Pearson’s correlation coefficient (*r*) was used to investigate relationships between variables in m.3243A>G patients with and without MELAS syndrome.

## Results

### Demographics and clinical characteristics

Participant demographics are summarized in Table 1, with individual data for m.3243A>G patients in Supplementary B Tables 1 and 2. Patients with MELAS syndrome had significantly lower body weight compared to non-MELAS patients (*P*  *<* 0.05). No significant differences were found between MELAS syndrome/non-MELAS patients for age or heteroplasmy levels in blood or urine (*P* > 0.05) (Table 1).

**Table 1:** Demographics for MELAS syndrome and non-MELAS m.3243A>G patients and age- and sex-matched controls

	MELAS syndrome (*n* = 6)	Non-MELAS (*n* = 10)	Healthy controls (*n* = 16)	*P*-value	Median difference	95% CI
Sex (male/female)	3/3	6/4	9/7	NA	NA	NA
Age (years)	30 (29.3–45)	37.5 (32.8–48.3)	35.5 (28.5–50.5)	0.918	−7.5	−20.5, 15
Height (m)	1.61 (1.59–1.65)	1.70 (1.63–1.74)	1.73 (1.68–1.79)	**0**.**025**	−0.09	−0.17, 0.03
Body weight (kg)	54.5 (48.5–58.1)*	68.7 (48.5–89.1)	78.5 (67.5–84.8)	**0**.**009**	−14.2	−38.6, −2.6
BMI (kg/m^2^)	21.3 (18.5–22.2)	24.3 (22.8–28)	25 (21.5–26.6)	0.121	−3	−10.5, 0.4
Serum lactate (mmol/L)	2.7 (1.7–3.6)	1.4 (1.2–2.3)^a^	NA	0.224	1.3	−0.7, 2.6
Systolic blood pressure (mmHg)	117 (113–125.5)	124.5 (116.5–134.3)	NA	0.385	−7.5	−22, 10
Diastolic blood pressure (mmHg)	79 (72–85.3)	82.5 (75.5–84)	NA	0.971	−3.5	−14, 8.5
Smoking history						
Never smoked, No.	4	4	NA	NA	NA	NA
Former smoker, No.	0	2	NA	NA	NA	NA
Current smoker, No.	0	2	NA	NA	NA	NA
Not available, No.	2	2	NA	NA	NA	NA
Occupational status						
Employed, No.	0	8	NA	NA	NA	NA
Not working, No.	5	2	NA	NA	NA	NA
Not available, No.	1	0	NA	NA	NA	NA
Disease severity—NMDAS						
I: Current function (0–50)	16 (15–17.8)*	5.5 (2–7.5)	NA	**0**.**015**	10.5	5.5, 16
II: System-specific involvement (0–45)	17.5 (15.5–20.3)*	10.5 (4.5–14.3)	NA	**0**.**012**	7	1.5, 15
III: Current clinical assessment (0–50)	10 (9–11.8)**	3.5 (1–7.3)	NA	**0**.**007**	6.5	1, 11.5
NMDAS (total score) (0–145)^b^	43 (35.6–51.2)**	23.4 (5.9–26.6)	NA	**0**.**008**	19.7	6.8, 42.3
Heteroplasmy						
Blood heteroplasmy (%)	26 (22–32)	24 (17–27)	NA	0.394	3.5	−5.5, 16
Age-corrected blood heteroplasmy (%)	73 (61–94)	70 (56–96)	NA	0.483	2.8	−3.3, 4.5
Urine heteroplasmy (%)	77 (61–86)	78 (58–84)	NA	0.785	2.5	−25.5, 25

Data are described as median (IQR) unless otherwise stated. Median difference = MELAS syndrome—non-MELAS. Bold denotes statistically significant values.

Significant difference between MELAS syndrome versus non-MELAS, *P < 0.05, **P < 0.01.

BMI, body mass index; CI, confidence interval; MELAS, mitochondrial encephalomyopathy with lactic acidosis and stroke-like episodes; NA, not-applicable.

^a^Missing data from two participants.

^b^Total NMDAS score is calculated by summing the scores obtained for each section (Sections I–III). Higher scores indicate greater disease seventy.

The number of stroke-like episode in MELAS syndrome patients ranged between one (*n* = 1 patient) and two episodes (*n* = 5 patients). Four of the six MELAS syndrome patients presented with the first stroke-like-episode before the age of 40 years (consistent with the original definition of MELAS), while two presented with their first stroke-like-episode above age 40. All MELAS syndrome participants had a documented history of seizures, with four taking regular anti-seizure medications at the time of the study. In contrast, none of the non-MELAS participants had a history of seizures. Common clinical features of mitochondrial disease included gastrointestinal disturbance (MELAS: 83%, non-MELAS: 90%), hearing impairment (MELAS: 100%, non-MELAS: 40%), diabetes mellitus (MELAS: 50%, non-MELAS: 45%), headaches/migraines (MELAS: 67%, non-MELAS: 60%), exercise intolerance (MELAS: 67%, non-MELAS: 50%) and gait instability (MELAS: 83%, non-MELAS: 40%) (Supplementary B Tables 1 and 2).

### Disease severity

**Figure 1:**
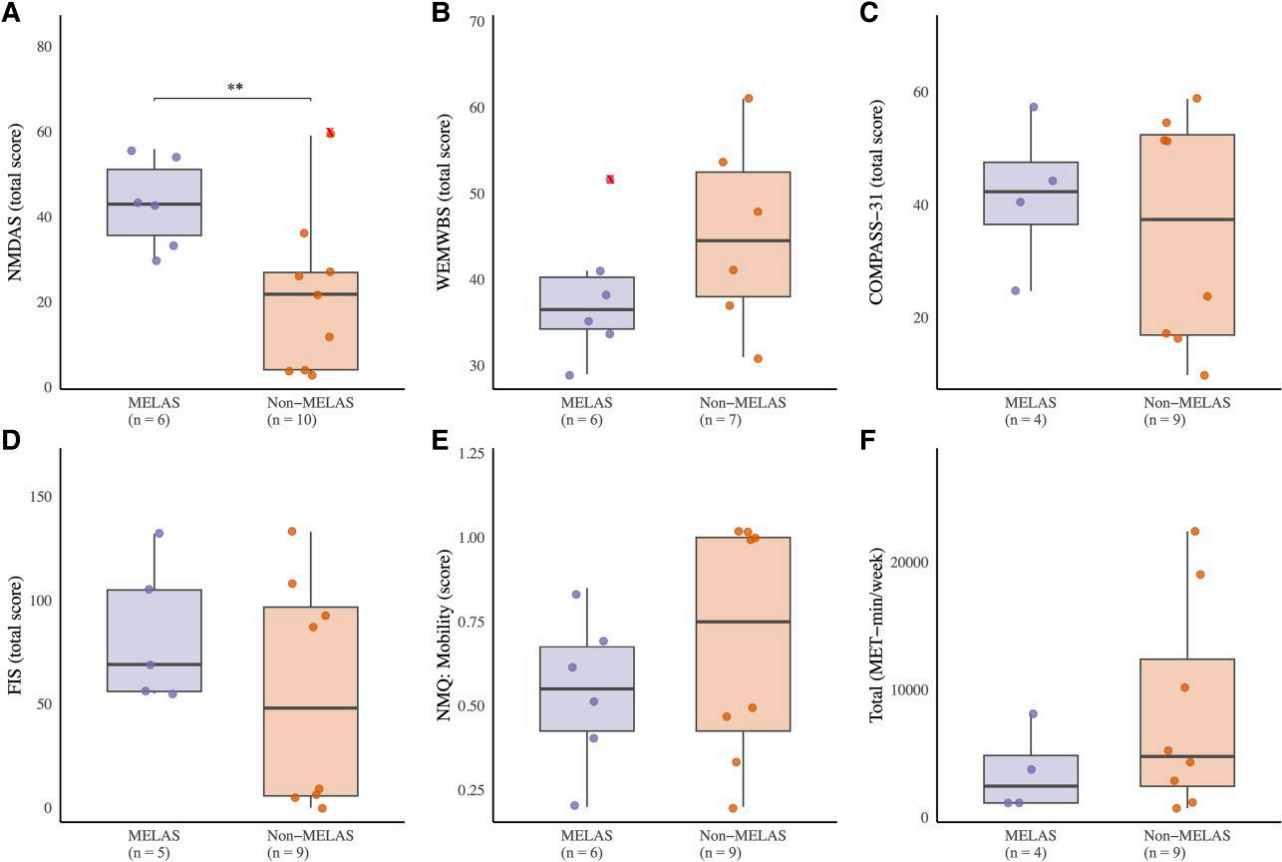
**Differences between m.3243A>G patient groups (MELAS syndrome versus. non-MELAS)—disease severity and PROs (A-F).** Left: (**A**) Disease severity (NMDAS); (**B**) mental well-being (WEMWBS); (**C**) self-reported autonomic function (COMPASS 31); (**D**) patient-reported fatigue (FIS); (**E**) health-related QoL (NMQ) mobility domain; and (**F**) self-reported physical activity (IPAQ). Data points represent individuals within the m.3243A>G MELAS syndrome and non-MELAS patient groups. Data presented as median, with whisker ends set at 1.5* IQR above the third quartile and 1.5*IQR below the first quartile. Minimum and maximum values outside this range are shown as outliers, x Maximum. Statistical significance was assessed using independent *t*-tests; significant difference between MELAS syndrome versus. non-MELAS, **P < 0.01. MELAS, mitochondrial encephalopathy, lactic acidosis and stroke-like episodes; MET, metabolic equivalent task.

Disease severity in m.3243A>G patients was assessed using the NMDAS, with scores categorized as mild (0–24), moderate (25–49) or severe (>50). Five patients had mild disease (all non-MELAS), eight patients had moderate disease (four non-MELAS, four MELAS syndrome) and three had severe disease (one non-MELAS and two MELAS syndrome). MELAS syndrome patients had a significantly higher disease burden (median [IQR], 43 [35.6–51.2] versus 23.4 [5.9–26.6], *P* < 0.01) compared to non-MELAS (Table 1 and Fig. 1A). A total of six patients had an NMDAS cognitive sub-score ≥ two (i.e. combined test percentiles ≤59th percentile). Of these, five (83%) had MELAS syndrome, compared to only one patient (10%) in the non-MELAS group (Supplementary B Table 1).

### Patient-reported outcomes

Despite a higher disease burden in patients with MELAS syndrome, there were no significant differences between MELAS syndrome/non-MELAS patients in fatigue impact (FIS), mental well-being (WEMWBS), health-related QoL (NMQ), autonomic function (COMPASS-31) or self-reported physical activity (IPAQ) (Table 2 and Fig. 1B–F). Severe (FIS ≥ 40) or excessive (FIS ≥ 80) fatigue was reported by all (*n* = 5) patients with MELAS syndrome and 50% (*n* = 5) of non-MELAS. In line with this, energy/fatigue was the lowest scoring domain of health-related QoL for both MELAS syndrome/non-MELAS patients (Table 2). Both MELAS syndrome/non-MELAS patients had notably lower WEMWBS scores (MELAS: 37 [34–40], non-MELAS: 41 [34–51]) compared to population norms (51 ± 7).^36^ Of those who completed the COMPASS-31 questionnaire, all MELAS syndrome patients (*n* = 4/4) and 67% (*n* = 6/9) non-MELAS patients had a score ≥20, indicative of moderate-to-severe autonomic dysfunction. With the exception of one patient with non-MELAS, all other patients were classified as having either high or moderate physical activity levels based on the IPAQ scoring criteria.

**Table 2:** PROs for MELAS syndrome and non-MELAS m.3243A>G patients

		*n*	MELAS syndrome^a^	*n*	Non-MELAS	Median difference	95% CI	*P-*value
Fatigue Impact Scale (FIS)	Cognitive functioning (0–40)	5	19 (17–27)	9	13 (2–23)	6	−11, 27	0.358
	Physical (0–40)	5	19 (18–24)	9	28 (1–30)	−9	−16, 38	0.446
	Psychosocial (0–80)	5	31 (26–45)	9	29 (3–41)	2	−15, 44.5	0.246
	FIS (total score) (0–160)^b^	5	69 (56–105)	9	77 (6–93)	−8	−37.5, 99.5	0.286
Health-related QOL—Newcastle Mitochondrial-QoL questionnaire (NMQ)^c^	Mobility (0–100)	6	55 (43–68)	9	50 (35–100)	5	−60, 35	0.530
	Activities of daily living (%)	6	88 (72–98)	9	100 (69–100)	−13	−31, 28	0.889
	Energy/fatigue (0–100)	6	31 (27–36)	9	31 (6–81)	0	−59, 31	0.401
	Vision (0–100)	6	75 (44–88)	9	63 (25–100)	13	−50, 63	0.720
	Communication (0–100)	6	52 (29–63)	9	83 (63–92)	−31	−67, 4	0.078
	Memory/cognition (0–100)	6	38 (25–50)	9	100 (25–100)	−63	−88, 25	0.167
	Food and digestion (0–100)	6	75 (64–86)	9	67 (63–92)	8	−33, 31	0.928
	Pain (0–100)	6	69 (36–78)	9	50 (25–100)	19	−75, 55	0.958
	Muscle stiffness (0–100)	6	66 (44–83)	9	44 (19–100)	22	−50, 59	0.796
	Migraine/headache (0–100)	6	71 (67–88)	9	50 (25–100)	21	−42, 63	0.765
	Emotional well-being (0–100)	6	46 (29–50)	9	58 (25–100)	−13	−75, 25	0.281
	Stigma (0–100)	6	54 (38–65)	9	75 (33–100)	−21	−67, 29	0.423
	Family role (0–100)	6	59 (41–73)	9	63 (35–100)	−3	−63, 37	0.461
	Personal relationships (0–100)	6	48 (43–59)	9	63 (38–100)	−15	−55, 19	0.184
	Social role/support (0–100)	6	54 (50–58)	9	50 (25–100)	4	−50, 33	0.461
	Diabetes (0–100)^d^	3	95 (78–98)	5	65 (10–70)	30	−10, 95	0.082
Mental well-being	WEMWBS (total) (14–70)^e^	6	37 (34–40)	7	41 (34–51)	−4.5	−19.5, 8.5	0.366
Autonomic function—composite Autonomic symptom score-31 (COMPASS 31)	Orthostatic intolerance (0–100)	4	16 (3–20)	9	18 (3–23)	−2	−20, 16	0.810
	Vasomotor (0–100)	4	0 (0–0.62)	9	0 (0–0)	0	−1.7, 1.3	0.574
	Secretomotor (0–100)	4	4.3 (0.5–6.4)	9	4.3 (0–8)	0	−6.4, 6.4	0.723
	Gastrointestinal (0–100)	4	5.4 (0.2–15.2)	9	8.5 (6.7–12.3)	−3.1	−11.2, 8.9	0.646
	Bladder (0–100)	4	1.7 (0–3.3)	9	1.1 (0.3–1.9)	0.6	−1.7, 2.8	0.906
	Pupillomotor (0–100)	4	1.7 (0.4–2.7)	9	1.5 (0.8–2.2)	0.2	−1.8, 2	0.930
	COMPASS (total) (0–100)^f^	4	42.3 (36.5–47.5)	9	37.6 (17.1–51.6)	4.7	−20.2, 33.7	0.529
Self-reported physical activity (IPAQ)^g^	Work-related PA (MET-min/week)	4	0 (0–0)	9	565 (0–5544)	−565	−13485, 0	0.070
	Transport-related PA (MET-min/week)	4	1122 (545–1683)	9	1188 (990–1233)	−66	−1035, 792	0.790
	Domestic and garden PA (MET-min/week)	4	480 (360–695)	9	300 (0–420)	180	−1010, 790	0.918
	Leisure-time PA (MET-min/week)	4	884 (467–2277)	9	0 (0–2628)	884	−2259, 5544	0.537
	Walking (MET-min/week)	4	1980 (668–4158)	9	1386 (1188–5148)	594	−4851, 5742	0.487
	Moderate (MET-min/week)	4	480 (360–695)	9	540 (0–1680)	−60	−1790, 790	0.277
	Vigorous (MET-min/week)	4	0 (0–120)	9	400 (0–2400)	−400	−6960, 240	0.109
	Total physical activity (MET-min/week)	4	2460 (1148–4853)	9	4326 (1188–10 170)	−1866	−13097, 4750	0.260

Data are described as median (IQR) unless otherwise stated. Median difference = MELAS syndrome—non-MELAS.

CI, confidence interval; MELAS, mitochondrial encephalomyopathy with lactic acidosis and stroke-like episodes; MET, metabolic equivalent task; WEMWBS, Warwick–Edinburgh mental wellbeing scale.

^a^Missing data reflect incomplete questionnaire responses by some participants.

^b^Sum of physical (17-items), cognitive (14-items) and psychosocial (9-items) sub-scales. Higher scores indicate a greater impact of fatigue on a person’s activities.

^c^Raw domain scores summed and transformed into a scale score, range from 0 to 100. Higher scores define a greater perceived QoL and health status.

^d^Completed only by participants for whom this was applicable.

^e^Sum of 14-items. Total scores range is from 14 to 70. Higher scores indicate greater positive mental well-being.

^f^Sum of six domains: orthostatic intolerance (4-items), vasomotor (3-items), secretomotor (4-items), gastrointestinal (12-items), bladder (3-items) and pupillomotor (5-item). Total weighted score range is from 0 to 100. Higher scores indicate higher autonomic dysfunction.

^g^MET minutes a week, where MET minutes represent the amount of energy expended carrying out physical activity. Rest is considered to expend 1 MET, walking is considered to be 3.3 METS, moderate physical activity is considered to be 4 METS and vigorous physical activity is considered to be 8 METS.

### Performance based outcome measures

#### Functional capacity

TUG times were similar between MELAS syndrome/non-MELAS patients (*P* > 0.05) (Table 3), with no patients scoring above the 13.5-second fall-risk threshold.^54^ Peak handgrip strength was lower than population norms^40^ for the majority of patients (100% MELAS syndrome, 70% non-MELAS), with no significant differences between MELAS syndrome/non-MELAS patients (*P* > 0.05) (Table 3).

**Table 3:** Functional/exercise capacity measures for MELAS syndrome and non-MELAS m.3243A>G patients

		*N*	MELAS syndrome^a^	*n*	Non-MELAS	Median difference	95% CI	*P*-value
Functional capacity	Timed-up and go (sec)	6	10.2 (8.4–11.5)	10	8.3 (7.2–10)	1.93	−1.5, 7.6	0.238
	Peak handgrip strength (kg)	5	23.1 (22–24)	10	32 (19.5–38)	−13.5	−63.5, 2	0.121
CPET with non-invasive cardiac output measurement	Resting heart rate (bpm)	5	85 (78–90)	10	69 (62–83)	16.3	−7.7, 44.4	0.239
	Resting stroke volume (L/min)	4	60.7 (53.8–68.4)	10	77.0 (73.8–84.1)	−17.3	−30.8, −2	0.065
	Resting stroke volume index (L/m²)	4	37.4 (32.4–44.8)	10	43.2 (38.4–46.6)	−5.8	−15.1, 11.3	0.738
	Resting cardiac output (L/min)	4	4.6 (4.2–5.1)	10	5.5 (4.9–6.4)	−0.9	−2.0, 0.5	0.110
	Resting cardiac index (L/m^2^)	4	3.1 (2.6–3.6)	10	2.9 (2.8–3.2)	0.1	−0.7, 0.7	0.975
	Resting VO_2_ (L/min)	5	0.18 (0.17–0.23)	10	0.29 (0.22–0.31)	−0.1	−0.13, 0.01	**0.018**
	Resting VO_2_ (ml/kg/min)	5	4.1 (3.3–4.3)	10	4.3 (3–4.7)	−0.2	−1.5, 1.4	0.941
	Peak heart rate (bpm)	5	152 (122–156)	10	134 (116–157)	18	−30, 44	0.679
	Peak stroke volume (L/min)	4	64.4 (58.6–67.8)***	10	108.6 (86–147.6)	−44.2	−96.0, −18.3	**<0.001**
	Peak stroke volume index (L/m^2^)	4	39.6 (35.4–44.1)*	10	54.1 (50–77)	−14.5	−45.5, 0.9	**0.019**
	Peak cardiac output (L/min)	4	8 (7–9.1)***	10	15.6 (14–16.5)	−7.6	−9.7, −4.3	**<0.001**
	Peak cardiac index (L/m^2^)	4	5.3 (4.2–6.3)*	10	8.2 (7.9–8.5)	−2.8	−4.5, 1.5	**0.012**
	Peak VO_2_ (L/min)	5	0.86 (0.81–0.89)*	10	1.26 (0.99–2.02)	−0.4	−1.42, −0.07	**0.011**
	Peak VO_2_ (ml/kg/min)	5	16.5 (13.8–20.3)	10	19.8 (13.6–26.6)	−3.4	−15.3, 6.3	0.196
	Anaerobic threshold (L/min)	5	0.48 (0.48–0.52)*	10	0.73 (0.61–1.17)	−0.25	−0.76, −0.06	**0.014**
	Anaerobic threshold (ml/kg/min)	5	9.2 (8.9–11.5)	10	11.7 (7.9–17)	−2.5	−9.4, 2.9	0.157
	Peak a-vO_2_ diff (ml/dL)	4	9.2 (8.9–11.5)	10	11.7 (7.9–17)	0.4	−5.1, 3.8	0.157
	Peak power (watts)	5	75 (68–78)*	10	90 (72–184)	−15	−132, 6	**0.041**
	Peak power (watts/kg)	5	1.3 (1.3–1.6)	10	1.3 (1–2.7)	0	−1.5, 0.7	0.266

Data are described as median (IQR) unless otherwise stated. Median difference = MELAS syndrome—non-MELAS. Bold denotes statistically significant values.

Significant difference between MELAS syndrome versus. non-MELAS, *P < 0.05, **P < 0.01, ***P < 0.001.

a-vO2 diff, arteriovenous oxygen difference; CI, confidence interval; CPET, cardiopulmonary exercise test; MELAS, mitochondrial encephalomyopathy with lactic acidosis and stroke-like episodes.

^a^Missing data reflect inability to complete the assessment in some participants.

#### Exercise capacity

**Figure 2:**
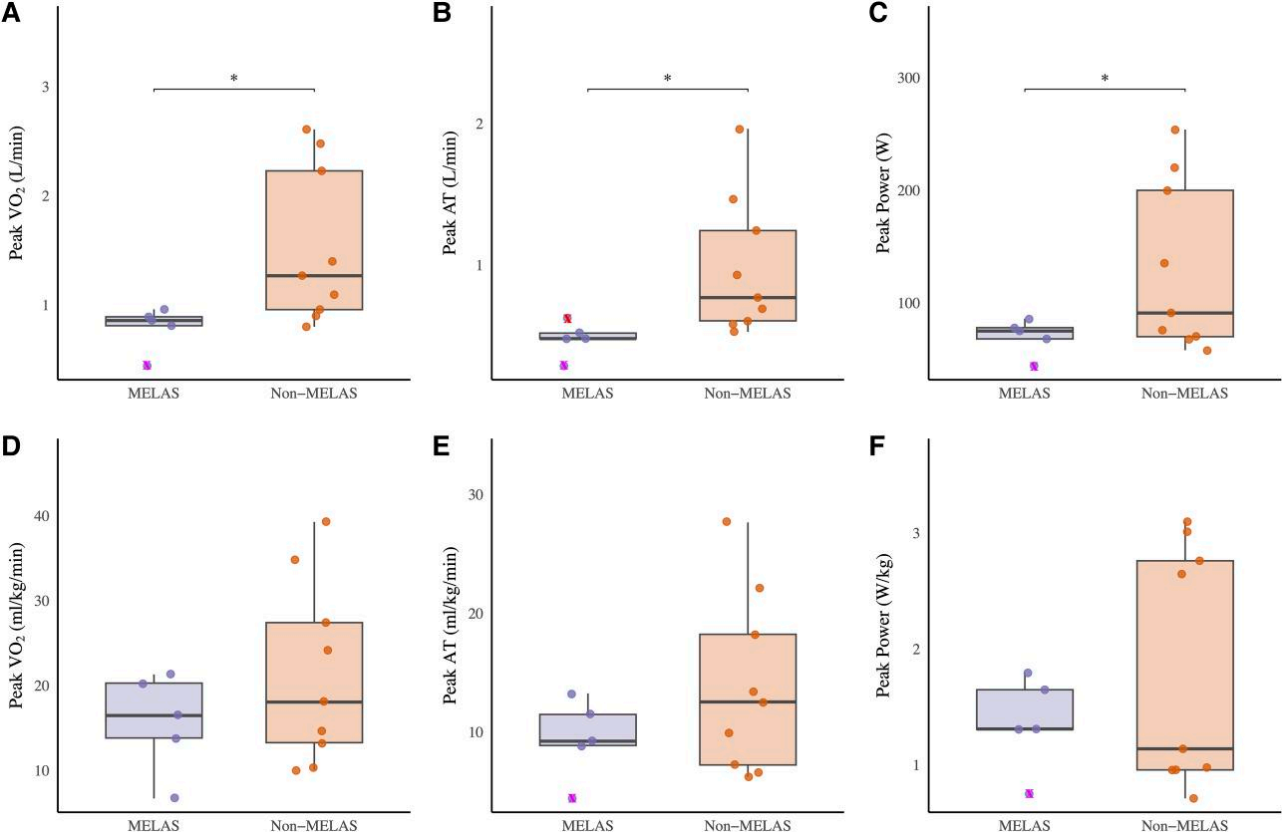
**Differences between m.3243A>G patient groups (MELAS syndrome versus non-MELAS)—measures of exercise capacity (A-F).** Left: (**A**) Absolute peak VO_2_ (L/min); (**B**) absolute anaerobic threshold (L/min); (**C**) absolute peak power (Watts [W]); (**D**) relative peak VO_2_ (ml/kg/min); (**E**) relative anaerobic threshold (ml/kg/min); and (**F**) relative peak power (W/kg). Data points represent individuals within the m.3243A>G MELAS syndrome (*n* = 5, purple) and non-MELAS (*n* = 10, orange) patient groups. Data presented as median, with whisker ends set at 1.5*IQR above the third quartile and 1.5*IQR below the first quartile. Minimum and maximum values outside this range are shown as outliers, x Minimum, x Maximum. Statistical significance was assessed using independent *t*-tests; significant difference between MELAS syndrome versus non-MELAS, *P < 0.05. MELAS, mitochondrial encephalopathy, lactic acidosis and stroke-like episodes.

Absolute measures of peak exercise performance, including VO_2_, anaerobic threshold and power were significantly lower in patients with MELAS syndrome (*P* < 0.05) (Table 3 and Fig. 2A–C), but no significant differences were found when adjusted for body weight (Fig. 2D–F). Patients with MELAS syndrome also had a lower peak cardiac output, cardiac index, stroke volume and stroke volume index (*P* < 0.05–0.001) (Table 3).

#### Accelerometry—physical activity, inactivity and sleep

**Figure 3:**
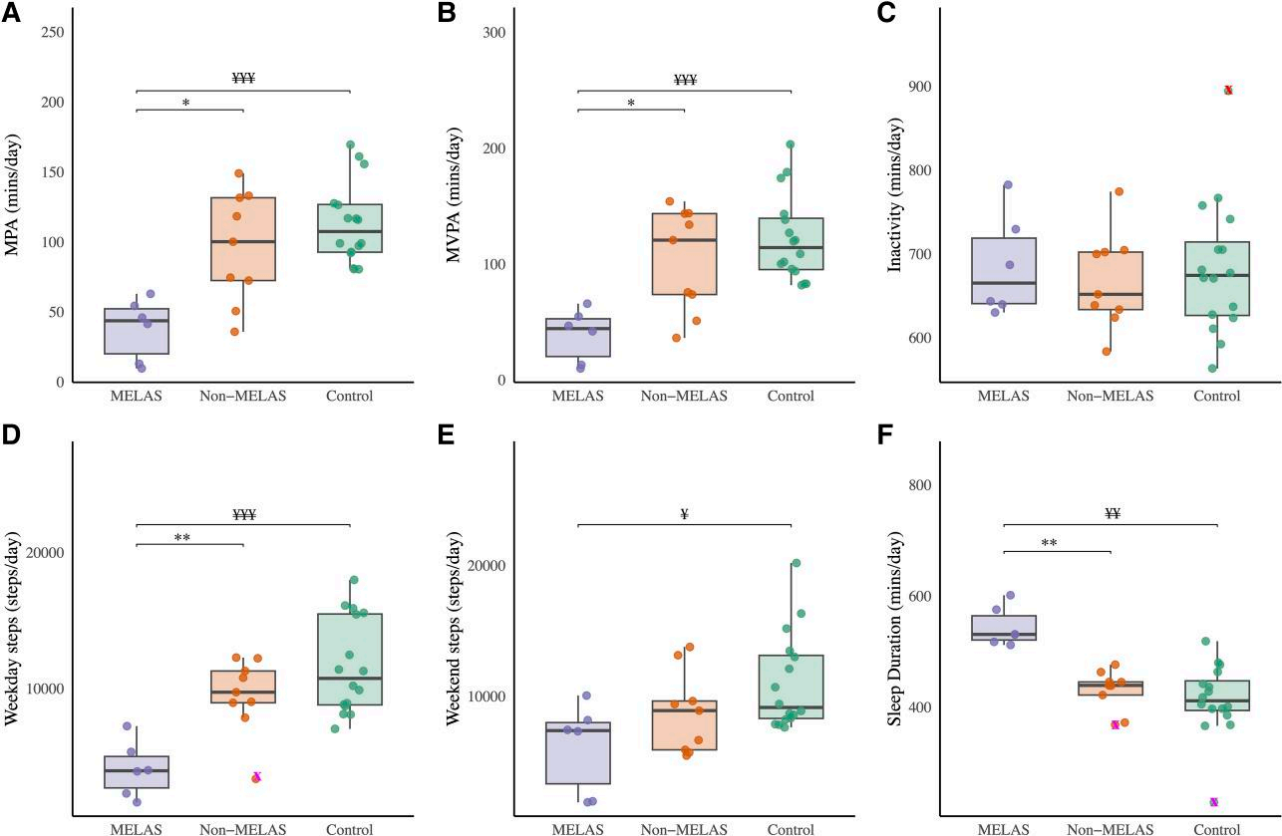
**Differences between m.3243A>G patient groups (MELAS syndrome versus non-MELAS) and controls—measures of accelerometry (A-F).** Left: (**A**) Total moderate physical activity (MPA) (min/day); (**B**) total MVPA (min/day); (**C**) total inactivity time (min/day); (**D**) weekday step count (steps/day); (**E**) weekend step count (steps/day); and (**F**) sleep duration (min/day). Data points represent individuals within the m.3243A>G MELAS syndrome (*n* = 6), non-MELAS (*n* = 9) and control groups (*n* = 16). Data presented as median, with whisker ends set at 1.5*IQR above the third quartile and 1.5*IQR below the first quartile. Minimum and maximum values outside this range are shown as outliers, x Minimum, x Maximum. Statistical significance was assessed using ANCOVA, adjusting for BMI. *Post-hoc* differences were identified using the Bonferroni test. Significant difference between MELAS syndrome versus. non-MELAS, *P < 0.05, *P* < 0.01 Significant difference between MELAS syndrome versus controls, ^¥^*P* < 0.05, ^¥¥^*P* < 0.01, ^¥¥¥^*P* < 0.001. MELAS, mitochondrial encephalopathy, lactic acidosis and stroke-like episodes.

Differences in acceleration categories between MELAS, non-MELAS and controls are shown in Table 4. Despite no difference in self-reported physical activity levels (IPAQ) between MELAS/non-MELAS, objective measurement shows patients with MELAS syndrome spent less time in moderate and MVPA compared to non-MELAS patients (*P* < 0.05), as well as controls (*P* < 0.001) (Fig. 3A and B). No significant differences were found in total light or vigorous physical activity, inactivity (Fig. 3C), or bouts of physical activity/inactivity (*P* > 0.05) (Table 4).

**Table 4:** Acceleration categories for MELAS syndrome and non-MELAS m.3243A>G patients and age- and sex-matched controls

	MELAS syndrome (*n* = 6)	Non-MELAS (*n* = 9)^a^	Healthy controls (*n* = 16)	*P*-value^b^	Median difference	95% CI
Waking hours (min/day)						
Inactivity	665 (641–719)	652 (634–702)	674 (627–714)	0.746	13	−63, 101
Light physical activity	116 (80–125)	183 (147–190)	150 (137–185)	**0.027**	−67	−126, −15
Moderate physical activity	44 (20–52)*,¥¥¥	100 (72–132)	107 (93–127)	**<0.001**	−56	−106, −14
Vigorous physical activity	0.5 (0.3–0.6)	2.3 (1.1–5.1)	4.2 (2.2–10.4)	0.261	−1.8	−9.9, 0.4
MVPA	44 (20–53)*,¥¥¥	121 (74–144)	114 (95–139)	**<0.001**	−76	−121, −13
Bouts of activity (min/day)						
Inactivity 30-min	510 (478–620)	406 (252–462)	447 (336–520)	0.080	104	10, 367
MVPA 5-min	1 (0.2–1.7)	8.2 (4.8–11)	9.2 (4.9–19.1)	0.205	−7.3	−11.4, 0.4
MVPA 10-min	0 (0–4.7)	1.9 (0–15.2)	9.9 (7.3–50.6)	0.079	−1.9	−17.5, 9.3
Steps/day						
Whole week	5552 (2906–6207)*,¥¥¥	9704 (7841–10 406)	10 069 (9360–12 179)	**<0.001**	−4152	−8066, −1551
Weekday	3954 (2695–5007)**,¥¥¥	9736 (8958–11 296)	10 752 (8802–15 489)	**<0.001**	−5781	−8833, −3339
Weekend	7383 (3317–7993)¥	8911 (5914–9641)	9148 (8311–13 128)	**0.019**	−1528	−7467, 2266
Sleep						
Sleep duration (min/day)	531 (521–565)**,¥¥	439 (422–445)	412 (394–447)	**<0.001**	92	69, 159
Sleep efficiency (%)	0.89 (0.86–0.92)	0.86 (0.85–0.88)	0.89 (0.86–0.90)	0.479	0.02	−0.03, 0.09

Data are described as median (IQR) unless otherwise stated. Median difference = MELAS syndrome—non-MELAS. Bold denotes statistically significant values.

Significant difference between MELAS syndrome versus non-MELAS, *P < 0.05, **P < 0.01,

Significant difference between MELAS syndrome versus healthy controls, ¥*P* < 0.05, ¥¥*P* < 0.01, ¥¥¥*P* < 0.001.

No significant differences were observed between non-MELAS and healthy controls.

BMI, body mass index; CI, confidence interval; MELAS, mitochondrial encephalomyopathy with lactic acidosis and stroke-like episodes; MVPA, moderate-vigorous physical activity.

^a^Missing data from one participant.

^b^Adjusted for BMI.

Patients with MELAS syndrome averaged significantly fewer steps per day than non-MELAS and controls (*P* < 0.05 and *P* < 0.001, respectively) (Table 4 and Fig. 3D). Weekend steps were lower in MELAS syndrome compared to controls (*P* < 0.05), but not compared to non-MELAS patients (Table 4 and Fig. 3E). Patients with MELAS syndrome had significantly longer sleep duration compared to non-MELAS and controls (*P* < 0.01), but no differences in sleep efficiency (Table 4 and Fig. 3F).

### Associations between outcome measures in MELAS syndrome and non-MELAS

#### Demographics

In both MELAS syndrome and non-MELAS patients, increasing age correlated with higher inactivity (*r*  *=* 0.87 to 0.91, *P*  *<* 0.05–0.01) (Supplementary B Tables 3–6). In MELAS syndrome, age also inversely correlated with VO_2peak_ (ml/kg/min) and cardiac index (*r*  *=* −0.1 to 0.90, *P*  *<* 0.05–0.01) (Supplementary B Tables 3 and 5). In non-MELAS patients, increasing age was associated with lower peak HR (*r*  *=* −0.69), higher peak SV/stroke volume index (SVI) (*r*  *=* 0.82 to 0.83), longer TUG time (*r*  *=* 0.71) and reduced physical activity (steps, 5-min MVPA) (*r*  *=* −0.71 to −0.81) (*P*  *<* 0.05–0.01) (Supplementary B Tables 4 and 6). Age also negatively correlated with well-being (WEMWBS) in non-MELAS (*r*  *=* −0.76), but positively with perceived energy/fatigue (NMQ) (*r*  *=* 0.84) in MELAS syndrome (*P*  *<* 0.05) (Supplementary B Tables 7 and 8).

BMI and body weight were not associated with outcomes in non-MELAS (Supplementary B Tables 4 and 6–8). In MELAS syndrome, BMI inversely correlated with peak cardiac index (*r*  *=* −0.97, *P* < 0.05) and higher body weight was associated with lower peak exercise capacity (VO_2_ (ml/kg/min), anaerobic threshold (ml/kg/min) and SVI) (*r*  *=* −0.89 to −0.98, *P* < 0.05–0.01), greater handgrip strength (*r*  *=* 0.89, *P* < 0.05) and poorer perceived mobility (NMQ) (*r*  *=* −0.91, *P* < 0.05) (Supplementary B Tables 3, 5, 7 and 8).

mtDNA blood heteroplasmy (age adjusted) was not associated with outcomes in MELAS syndrome (Supplementary B Tables 3, 5, 7 and 8). In non-MELAS, higher heteroplasmy correlated with lower moderate and MVPA (*r*  *=* −0.77 to −0.90, *P*  *<* 0.05–0.01), longer sleep (*r* = 0.97) (Supplementary B Tables 4 and 6), poorer perceived mobility and ADLs (NMQ) (*r*  *=* −0.74 to −0.77, *P* < 0.05) and lower self-reported physical activity (IPAQ: walking, moderate and total) (*r*  *=* −0.72 to −0.92, *P* < 0.05–0.01) (Supplementary B Tables 7 and 8).

#### Disease severity, fatigue and performance outcome measures

**Figure 4:**
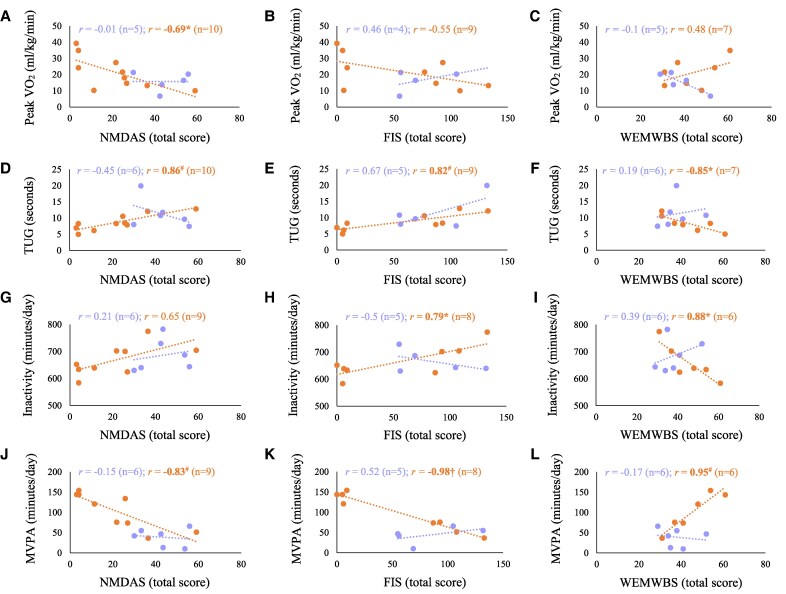
**Pearsons’s correlation plots showing relationships between outcome measures in m.3243A>G patient groups (MELAS syndrome and non-MELAS)** left: disease severity (NMDAS) correlated with (from top to bottom): (**A**) peak VO_2_ (ml/kg/min), (**D**) TUG (seconds), (**G**) total inactivity (min/day) and (**J**) total MVPA (min/day). Patient-reported fatigue (FIS) correlated with (from top to bottom): (**B**) peak VO_2_ (ml/kg/min), (**E**) TUG (seconds), (**H**) total inactivity (min/day) and (**K**) total MVPA (min/day). Mental well-being (WEMWBS) correlated with (from top to bottom): (**C**) peak VO_2_ (ml/kg/min), (**F**) TUG (seconds), (**I**) total inactivity (min/day) and (**L**) total MVPA (min/day). Data points represent individuals within the m.3243A>G MELAS syndrome and non-MELAS patient groups. Sample size (*n*) and Pearson’s correlation coefficients (*r*) are shown for each correlation. Statistically significant correlations are denoted in bold, *P < 0.05, ^#^*P* < 0.01 and ^†^*P* < 0.001. MELAS, mitochondrial encephalopathy, lactic acidosis and stroke-like episodes.

In MELAS syndrome, disease severity (NMDAS) and fatigue (FIS) showed minimal associations with performance measures (Fig. 4A, B, D, E, G, H, J and K), except for a positive correlation between fatigue and MVPA-5 min bouts (*r* = 0.88, *P*  *<* 0.05) (Supplementary B Tables 3 and 5).

In contrast, in non-MELAS, both NMDAS and FIS correlated with multiple PerfOs, including negative correlations with peak HR, a-vO_2_ diff, handgrip strength and moderate and MVPA (*r*  *=* −0.64 to −0.98, *P*  *<* 0.05–0.001) (Fig. 4J and K) and positive correlations with peak SV/SVI and TUG time (*r*  *=* 0.67 to 0.86, *P* < 0.05–0.01) (Fig. 4D and E). NMDAS also negatively correlated with peak VO_2_ (Fig. 4A) and anaerobic threshold (ml/kg/min) and peak power (W/kg) (*r*  *=*  *−*0.63 to −0.71, *P* < 0.05), while FIS also correlated with higher inactivity levels (*r*  *=* 0.73 to 0.79, *P* < 0.05) (Fig. 4H) and lower daily steps (*r* = 0.87, *P<* 0.01) (Supplementary B Tables 4 and 6).

#### Disease severity, fatigue and patient-reported outcomes

In non-MELAS, disease severity (NMDAS) positively correlated with fatigue (FIS) (*r*  *=* 0.86, *P* < 0.01) (Supplementary B Tables 4 and 6), and both were inversely correlated with well-being (WEMWBS), perceived mobility and energy/fatigue (NMQ) (*r* = **−**0.69 to −0.96, *P* < 0.05–0.001) and positively associated with autonomic dysfunction (COMPASS) (*r* = 0.85 to 0.96, *P* < 0.01–0.001) (Supplementary B Tables 7 and 8). In contract, MELAS syndrome patients showed no significant association between disease severity and PROs, with the exception of fatigue being inversely correlated with ADL (*r*  *=*  **−**0.98, *P* < 0.01) (Supplementary B Tables 3, 5, 7 and 8).

#### Associations between PerfOs and PROs

In non-MELAS patients, well-being (WEMWBS), perceived mobility and energy/fatigue (NMQ) all positively correlated with several PerfO, including peak HR, handgrip strength and physical activity (moderate and MVPA, steps) (*r*  *=* 0.69 to 0.95, *P* < 0.05–0.001). These PROs all inversely correlated with TUG time (Fig. 4F**)** and peak SVI (*r*  *=* −0.68 to −0.85, *P* < 0.05–0.01), while well-being and mobility also inversely correlated with inactivity outcomes (*r*  *=* −0.73 to −0.91, *P* < 0.05) (Supplementary B Tables 7 and 8 and Fig. 4I).

The only shared significant relationships between PROs and PerfO in both MELAS syndrome and non-MELAS patients were positive correlations between well-being (WEMWBS) and MVPA-10 min (*r*  *=* 0.87 to 0.92, *P* < 0.05–0.01) (Supplementary B Tables 7 and 8). In MELAS syndrome patients, PerfO–PRO associations were fewer and often in the opposite direction to expected relationships. For instance, well-being (WEMWBS) inversely correlated with peak VO_2_ and anaerobic threshold (ml/kg/min) (*r*  *=* −0.88 to −0.95, *P* < 0.05); perceived mobility (NMQ) inversely correlated with MVPA-10mins (*r*  *=* −0.84, *P* < 0.05); and energy/fatigue inversely correlated with anaerobic threshold (ml/kg/min) (*r*  *=* −0.94, *P* < 0.05) (Supplementary B Tables 7 and 8).

Neither the MELAS syndrome nor non-MELAS patient groups demonstrated strong correlations between objectively measured and self-reported (IPAQ) physical activity measures (Supplementary B Tables 7 and 8). In non-MELAS, walking correlated with higher handgrip strength and greater light physical activity (*r*  *=* 0.69 to 0.81), vigorous activity with peak cardiac output (*r*  *=* 0.68) and total physical activity correlated with peak power (W/kg) (*r*  *=* 0.67) (*P* < 0.05). Additionally, sleep duration inversely correlated with moderate and total self-reported physical activity (*r*  *=*  *−*0.74 to −0.89) (*P* < 0.05 to 0.01). In MELAS syndrome, vigorous physical activity positively correlated with 5-min bout MVPA time (*r*  *=* 0.99, *P* < 0.05) and counterintuitively, with longer TUG time (*r*  *=* 0.98, *P* < 0.05).

Within-PRO associations also differed between MELAS/non-MELAS. While well-being (WEMWBS) was positively associated with energy/fatigue (NMQ) in both MELAS syndrome and non-MELAS (*r*  *=* 0.84 to 0.95, *P* < 0.05 to 0.01), the association between perceived mobility and well-being was positive in non-MELAS (*r*  *=* 0.93, *P* < 0.01), yet inversely correlated in MELAS syndrome (*r*  *=*  *−*0.86, *P* < 0.05). Additionally, autonomic dysfunction (COMPASS-31) was inversely associated with perceived mobility, energy/fatigue and ADLs (NMQ) in non-MELAS (*r*  *=*  *−*0.71 to −0.89, *P* < 0.05 to 0.0–1), while no such associations were observed in MELAS syndrome patients (*P* > 0.05).

## Discussion

This study conducted a comprehensive phenotypic comparison between m.3243A>G patients manifesting with MELAS syndrome and those without, with a focus on disease severity, PROs, objective performance measures and the relationships between these domains. Despite no difference in age or mtDNA heteroplasmy levels, MELAS syndrome patients exhibited greater disease burden, reduced exercise capacity and lower objectively measured physical activity levels. However, PROs were comparable across MELAS syndrome/non-MELAS, with a dissociation between subjective and objective assessments, particularly evident in patients with MELAS syndrome.

Previous studies have shown that m.3243A>G-related disease severity correlates with higher mtDNA heteroplasmy levels and age.^55-59^ However, MELAS syndrome patients in this study had significantly higher disease burden despite comparable mtDNA heteroplasmy and age. Patients with MELAS syndrome also had a lower body weight when compared to non-MELAS patients, consistent with previous studies.^59-61^ Although heteroplasmy has been associated with aerobic capacity in previous studies,^16,62,63^ age-adjusted blood mtDNA heteroplasmy had no relationship to outcomes in MELAS syndrome. In contrast, heteroplasmy in non-MELAS patients correlated with lower physical activity and poorer health-related QoL, indicating that heteroplasmy may have differential phenotype-specific value. While familial relationships have not been explicitly stated due to maintaining anonymity of participants, we observed considerable clinical heterogeneity among related individuals, suggesting that heteroplasmy alone does not fully explain phenotypic expression.

Despite a higher disease burden and reduced physical function, self-reported measures of fatigue, well-being, health-related QoL, autonomic symptoms and physical activity did not differ significantly from non-MELAS patients. This discordance suggests that perceived impact may not fully reflect clinical severity in m.3243A>G mitochondrial disease, particularly in MELAS syndrome. In non-MELAS, both disease severity and fatigue consistently correlated with poorer well-being, and health-related QoL, and greater autonomic dysfunction, as well as lower peak exercise performance and physical activity. However, in MELAS syndrome patients, these relationships were either minimal, absent or inverse, potentially reflecting altered disease perception which may reflect disease-related adaptation. Other studies have shown that QoL and fatigue only partially reflect clinical parameters.^14^ While prior mitochondrial disease research links disease severity to self-reported fatigue,^11,14,64^ this study observed this relationship only in non-MELAS patients, highlighting potential differences in how MELAS syndrome/non-MELAS patients perceive the impact of their condition. Of note, our NMDAS data also showed that patients with MELAS syndrome had greater cognitive impairment compared to non-MELAS patients, potentially contributing to this dissociation in perception.

Subjective-objective associations diverged further when comparing PRO–PerfO correlations. In non-MELAS patients, better well-being, health-related QoL and lower fatigue were associated with greater exercise performance and objectively measured physical activity levels. However, in MELAS syndrome these associations were largely lacking or reversed, with higher well-being and perceived energy/fatigue correlating with lower PerfOs. Additionally, although age was associated with a decline in some measures of physical performance in both MELAS syndrome/non-MELAS, the perception of these declines differed—increasing age was associated with higher perceived energy/fatigue in MELAS syndrome, while in non-MELAS, it was associated with poorer well-being. These findings reinforce the idea that self-reported measures alone may not accurately reflect physiological or functional status in MELAS syndrome and should be supplemented with objective measures.

Similar dissociation between PROs and objective function has been observed in other progressive diseases. In Duchenne muscular dystrophy, for example, patients often report high emotional wellbeing despite substantial physical decline.^65^ This ‘disability paradox’ may reflect a response shift, whereby individuals adapt their internal standards and perceptions in response to changes in health status.^66^ This discrepancy may be further amplified in individuals with cognitive impairment; in Huntington disease, for example, the psychometric reliability of PROs decrease with cognitive decline.^67^ Our findings highlight the importance of interpreting PROs as complementary, rather than surrogate endpoints in both research and clinical settings.

Exercise capacity was markedly lower in MELAS syndrome patients compared to non-MELAS in absolute terms but was similar when adjusted for body weight, highlighting the need for future studies to account for body weight and body composition.^68,69^ Nevertheless, both MELAS syndrome/non-MELAS patients had VO_2peak_ values considerably below reference standards,^70^ consistent with prior findings.^16,42,62,71-76^ Maximal oxygen consumption (VO_2_) reflects the product of maximum cardiac output (i.e. capacity of the cardiovascular system to deliver oxygen) and arterio-venous oxygen difference (i.e. ability of muscle to extract and utilize available oxygen). MELAS syndrome patients exhibited a lower peak cardiac output and cardiac index attributable to a reduced peak SV and SVI, while peak a-vO_2_ difference was similar between patients with MELAS syndrome/non-MELAS, suggesting a cardiovascular impairment rather than impaired muscle oxidative capacity.

Similarly, despite similar levels of self-reported physical activity, patients with MELAS syndrome demonstrated significantly lower objectively measured physical activity compared to both non-MELAS patients and healthy controls, with limited correlation between PROs and accelerometry. Our findings reinforce prior observations that self-reported physical activity can misestimate actual physical activity levels.^77^

Our findings align with a previous report showing lower objective physical activity levels in mitochondrial disease compared to matched controls.^17^ In the current study, MELAS syndrome patients completed approximately twice as many steps on the weekend compared to during the week, while non-MELAS patients maintained similar activity levels throughout the week. Given that all MELAS syndrome patients were unemployed, a ‘weekend warrior’ activity pattern potentially reflects reliance on caregiver support whereby informal caregivers were more likely to be off work themselves at the weekend and could support patients with activities. Although there was no difference in sleep efficiency between MELAS syndrome/non-MELAS, sleep duration was higher in MELAS syndrome patients, possibly reflecting symptoms such as seizures and stroke-like episodes, or sedative side effects of anti-seizure medication. However, sleep studies in m.3243A>G are limited,^78,79^ supporting the need for further studies to better understand this relationship.

The NMDAS^33^ is well-established for clinical monitoring, but was not designed for use in interventional trials. Composite tools, such as the mitochondrial myopathy composite assessment tool (MM-COAST), have been developed to provide integrated assessments of function and symptom burden.^80^ However, no analogous outcome measure currently exists for MELAS syndrome. Although not evaluated in this study, our findings suggest that MELAS-specific tools that combine PROs with objective, functional metrics may better capture disease impact and improve sensitivity in clinical trials. To contextualize this need, we assembled a comprehensive and up-to-date summary of all registered clinical trials in primary mitochondrial disease, including their primary outcomes and current status, which is provided in Supplementary A.

This study has several limitations. The small sample size reflects the rarity of MELAS syndrome and limits statistical power. Accordingly, the findings should be interpreted as preliminary pilot data rather than definitive. As a cross-sectional design, causal inferences are limited. Detailed cognitive testing was not performed, which limits interpretation of our findings. Magnetic resonance spectroscopy or measurement of cerebrospinal fluid lactate level was not performed, which would provide additional metabolic insights. Future studies should include larger, more diverse cohorts with longitudinal follow-up to track disease progression more accurately. Detailed body composition data should be incorporated to understand its role in physical performance and exercise capacity. Further exploration of physical activity patterns, alongside psychological and social factors, could clarify discrepancies between self-reported outcomes and objective measures, particularly in MELAS. Research on sleep patterns and disease severity in m.3243A>G is also needed. Incorporating new technologies and biomarkers would enhance disease monitoring and improve patient care.

Our findings reinforce the need for multi-modal outcome measures in MELAS syndrome. While PROs provide essential insights into patient experience, they may underestimate the true burden of disease in this population. Performance-based outcomes showed clearer differentiation between MELAS syndrome and non-MELAS and may offer greater sensitivity to functional impairment. As regulators increasingly emphasize patient-centred outcomes in rare disease trials,^31,81^ it is crucial that these are interpreted alongside objective performance data, particularly in complex conditions like MELAS syndrome. Future efforts should prioritize the development and validation of MELAS syndrome-specific composite outcome measures to ensure accurate, comprehensive and clinically meaningful evaluation of therapeutic interventions.

## Supplementary Material

fcaf342_Supplementary_Data

## Data Availability

The data reporting findings of this study are available from the corresponding author, upon reasonable request, whilst maintaining anonymization of participants.

## References

[1] Gorman GS, Chinnery PF, DiMauro S, et al Mitochondrial diseases. Nat Rev Dis Primers. 2016;2(1):16080. 27775730 10.1038/nrdp.2016.80

[2] Nesbitt V, Pitceathly RD, Turnbull DM, et al The UK MRC mitochondrial disease patient cohort study: Clinical phenotypes associated with the m.3243A>G mutation–implications for diagnosis and management. J Neurol Neurosurg Psychiatry. 2013;84(8):936–938. 10.1136/jnnp-2012-303528 23355809

[3] Gorman GS, Schaefer AM, Ng Y, et al Prevalence of nuclear and mitochondrial DNA mutations related to adult mitochondrial disease. Ann Neurol. 2015;77(5):753–759. 25652200 10.1002/ana.24362 PMC4737121

[4] Kaufmann P, Engelstad K, Wei Y, et al Natural history of MELAS associated with mitochondrial DNA m.3243A>G genotype. Neurology. 2011;77(22):1965–1971. 10.1212/WNL.0b013e31823a0c7f PMC3235358 22094475

[5] Ng YS, Bindoff LA, Gorman GS, et al Consensus-based statements for the management of mitochondrial stroke-like episodes. Wellcome Open Res. 2019;4:201. 32090171 10.12688/wellcomeopenres.15599.1 PMC7014928

[6] Mancuso M, Orsucci D, Angelini C, et al The m.3243A>G mitochondrial DNA mutation and related phenotypes. A matter of gender? J Neurol. 2014;261(3):504–510. 10.1007/s00415-013-7225-3 24375076

[7] Chinnery PF, Zwijnenburg PJ, Walker M, et al Nonrandom tissue distribution of mutant mtDNA. Am J Med Genet. 1999;85(5):498–501. 10405450

[8] Frederiksen AL, Andersen PH, Kyvik KO, Jeppesen TD, Vissing J, Schwartz M. Tissue specific distribution of the 3243A->G mtDNA mutation. J Med Genet. 2006;43(8):671–677. 16490799 10.1136/jmg.2005.039339 PMC2564591

[9] Tinker RJ, Lim AZ, Stefanetti RJ, McFarland R. Current and emerging clinical treatment in mitochondrial disease. Mol Diagn Ther. 2021;25(2):181–206. 33646563 10.1007/s40291-020-00510-6 PMC7919238

[10] Bergs PMJ, Maas DM, Janssen MCH, Groothuis JT. Feasible and clinical relevant outcome measures for adults with mitochondrial disease. Mol Genet Metab. 2022;135(1):102–108. 34961688 10.1016/j.ymgme.2021.12.010

[11] Gorman GS, Elson JL, Newman J, et al Perceived fatigue is highly prevalent and debilitating in patients with mitochondrial disease. Neuromuscul Disord. 2015;25(7):563–566. 26031904 10.1016/j.nmd.2015.03.001 PMC4502433

[12] Mancuso M, Angelini C, Bertini E, et al Fatigue and exercise intolerance in mitochondrial diseases. Literature revision and experience of the Italian network of mitochondrial diseases. Neuromuscul Disord. 2012;22:S226–S229. 23182644 10.1016/j.nmd.2012.10.012 PMC3526786

[13] Parikh S, Galioto R, Lapin B, et al Fatigue in primary genetic mitochondrial disease: No rest for the weary. Neuromuscul Disord. 2019;29(11):895–902. 31653361 10.1016/j.nmd.2019.09.012

[14] Verhaak C, de Laat P, Koene S, et al Quality of life, fatigue and mental health in patients with the m.3243A>G mutation and its correlates with genetic characteristics and disease manifestation. Orphanet J Rare Dis. 2016;11:25. 10.1186/s13023-016-0403-5 PMC4797235 26988355

[15] Thomas RH, Hunter A, Butterworth L, et al Research priorities for mitochondrial disorders: Current landscape and patient and professional views. J Inherit Metab Dis. 2022;45(4):796–803. 35543492 10.1002/jimd.12521 PMC9429991

[16] Taivassalo T, Dysgaard Jensen T, Kennaway N, DiMauro S, Vissing J, Haller RG. The spectrum of exercise tolerance in mitochondrial myopathies: A study of 40 patients. Brain. 2003;126(Pt 2):413–423. 12538407 10.1093/brain/awg028

[17] Apabhai S, Gorman GS, Sutton L, et al Habitual physical activity in mitochondrial disease. PLoS One. 2011;6(7):e22294. 21799815 10.1371/journal.pone.0022294 PMC3142121

[18] Taivassalo T, Haller RG. Implications of exercise training in mtDNA defects–use it or lose it? Biochim Biophys Acta. 2004;1659(2–3):221–231. 15576055 10.1016/j.bbabio.2004.09.007

[19] Shephard RJ, Allen C, Benade AJ, et al The maximum oxygen intake. An international reference standard of cardiorespiratory fitness. Bull World Health Organ. 1968;38(5):757–764. 5303329 PMC2554684

[20] Andrew P, Paul B, Joao C, et al ARTP statement on cardiopulmonary exercise testing 2021. BMJ Open Respir Res. 2021;8(1):e001121. 10.1136/bmjresp-2021-001121 PMC8593741 34782330

[21] Rosenlund M, Kinnunen U-M, Saranto K. The use of digital health services among patients and citizens living at home: Scoping review. J Med Internet Res. 2023;25:e44711. 36972122 10.2196/44711 PMC10131924

[22] Mittermaier M, Venkatesh KP, Kvedar JC. Digital health technology in clinical trials. NPJ Digit Med. 2023;6(1):88. 37202443 10.1038/s41746-023-00841-8 PMC10195788

[23] Masanneck L, Gieseler P, Gordon WJ, Meuth SG, Stern AD. Evidence from ClinicalTrials.gov on the growth of digital health technologies in neurology trials. NPJ Digit Med. 2023;6(1):23. 36765123 10.1038/s41746-023-00767-1 PMC9918454

[24] Barker J, Smith Byrne K, Doherty A, et al Physical activity of UK adults with chronic disease: Cross-sectional analysis of accelerometer-measured physical activity in 96 706 UK biobank participants. Int J Epidemiol. 2019;48(4):1167–1174. 30721947 10.1093/ije/dyy294 PMC6693885

[25] Jimenez-Moreno AC, Newman J, Charman SJ, et al Measuring habitual physical activity in neuromuscular disorders: A systematic review. J Neuromuscul Dis. 2017;4(1):25–52. 28269791 10.3233/JND-160195 PMC5345641

[26] Chandrasekaran R, Katthula V, Moustakas E. Patterns of use and key predictors for the use of wearable health care devices by US adults: Insights from a national survey. J Med Internet Res. 2020;22(10):e22443. 33064083 10.2196/22443 PMC7600024

[27] Liu F, Wanigatunga AA, Schrack JA. Assessment of physical activity in adults using wrist accelerometers. Epidemiol Rev. 2022;43(1):65–93. 34215874 10.1093/epirev/mxab004 PMC8900289

[28] van Eijk RPA, Bakers JNE, Bunte TM, de Fockert AJ, Eijkemans MJC, van den Berg LH. Accelerometry for remote monitoring of physical activity in amyotrophic lateral sclerosis: A longitudinal cohort study. J Neurol. 2019;266(10):2387–2395. 31187191 10.1007/s00415-019-09427-5 PMC6765690

[29] Rodríguez-Molinero A, Samà A, Pérez-López C, et al Analysis of correlation between an accelerometer-based algorithm for detecting parkinsonian gait and UPDRS subscales. Front Neurol. 2017;8:431. 28919877 10.3389/fneur.2017.00431 PMC5585138

[30] World Medical Association . World medical association declaration of Helsinki: Ethical principles for medical research involving human subjects. JAMA. 2013;310(20):2191–2194. 24141714 10.1001/jama.2013.281053

[31] U.S. Department of Health and Human Services Food and Drug Administration Center for Drug Evaluation and Research (CDER) Center for Biologics Evaluation and Research (CBER). Patient-focused drug development: Collecting comprehensive and representative input. Guidance for Industry, FDA Staff, and Other Stakeholders. FDA.gov; 2020.

[32] de Laat P, Janssen MC, Alston CL, Taylor RW, Rodenburg RJ, Smeitink JA. Three families with ‘de novo’ m.3243A>G mutation. BBA Clin. 2016;6:19–24. 27331024 10.1016/j.bbacli.2016.04.007 PMC4900294

[33] Schaefer AM, Phoenix C, Elson JL, McFarland R, Chinnery PF, Turnbull DM. Mitochondrial disease in adults: A scale to monitor progression and treatment. Neurology. 2006;66(12):1932–1934. 16801664 10.1212/01.wnl.0000219759.72195.41

[34] Fisk JD, Ritvo PG, Ross L, Haase DA, Marrie TJ, Schlech WF. Measuring the functional impact of fatigue: Initial validation of the fatigue impact scale. Clin Infect Dis. 1994;18(Suppl 1):S79–S83. 8148458 10.1093/clinids/18.supplement_1.s79

[35] Elson JL, Cadogan M, Apabhai S, et al Initial development and validation of a mitochondrial disease quality of life scale. Neuromuscul Disord. 2013;23(4):324–329. 23433484 10.1016/j.nmd.2012.12.012 PMC3841574

[36] Tennant R, Hiller L, Fishwick R, et al The Warwick-Edinburgh mental well-being scale (WEMWBS): Development and UK validation. Health Qual Life Outcomes. 2007;5(1):63. 18042300 10.1186/1477-7525-5-63 PMC2222612

[37] Sletten DM, Suarez GA, Low PA, Mandrekar J, Singer W. COMPASS 31: A refined and abbreviated composite autonomic symptom score. Mayo Clin Proc. 2012;87(12):1196–1201. 23218087 10.1016/j.mayocp.2012.10.013 PMC3541923

[38] Craig CL, Marshall AL, Sjöström M, *et al*. International physical activity questionnaire: 12-country reliability and validity. *Med Sci Sports Exerc*. 2003;35:1381-1395. 10.1249/01.MSS.0000078924.61453.FB 12900694

[39] Podsiadlo D, Richardson S. The timed “up & go”: A test of basic functional mobility for frail elderly persons. J Am Geriatr Soc. 1991;39(2):142–148. 1991946 10.1111/j.1532-5415.1991.tb01616.x

[40] Massy-Westropp NM, Gill TK, Taylor AW, Bohannon RW, Hill CL. Hand grip strength: Age and gender stratified normative data in a population-based study. BMC Res Notes. 2011;4(1):127. 21492469 10.1186/1756-0500-4-127 PMC3101655

[41] Beaver WL, Wasserman K, Whipp BJ. A new method for detecting anaerobic threshold by gas exchange. J Appl Physiol (1985). 1986;60(6):2020–2027. 3087938 10.1152/jappl.1986.60.6.2020

[42] Bates MG, Newman JH, Jakovljevic DG, et al Defining cardiac adaptations and safety of endurance training in patients with m.3243A>G-related mitochondrial disease. Int J Cardiol. 2013;168(4):3599–3608. 23742928 10.1016/j.ijcard.2013.05.062 PMC3819621

[43] van Hees VT, Gorzelniak L, Dean León EC, et al Separating movement and gravity components in an acceleration signal and implications for the assessment of human daily physical activity. PLoS One. 2013;8:e61691. 23626718 10.1371/journal.pone.0061691 PMC3634007

[44] Migueles JH, Rowlands AV, Huber F, Sabia S, Hees V. GGIR: A research community–driven open source R package for generating physical activity and sleep outcomes from multi-day raw accelerometer data. J Meas Phys Behav. 2019;2(3):188.

[45] RCoreTeam . R: A language and environment for statistical computing. R Foundation for Statistical Computing. ISBN 3-900051-07-0. http://www.R-project.org/

[46] Charman SJ, van Hees VT, Quinn L, et al The effect of percutaneous coronary intervention on habitual physical activity in older patients. BMC Cardiovasc Disord. 2016;16(1):248. 27912733 10.1186/s12872-016-0428-7 PMC5135787

[47] van Hees VT, Fang Z, Langford J, et al Autocalibration of accelerometer data for free-living physical activity assessment using local gravity and temperature: An evaluation on four continents. J Appl Physiol (1985). 2014;117(7):738–744. 25103964 10.1152/japplphysiol.00421.2014 PMC4187052

[48] da Silva IC, van Hees VT, Ramires VV, et al Physical activity levels in three Brazilian birth cohorts as assessed with raw triaxial wrist accelerometry. Int J Epidemiol. 2014;43(6):1959–1968. 25361583 10.1093/ije/dyu203 PMC4276065

[49] Sabia S, van Hees VT, Shipley MJ, et al Association between questionnaire- and accelerometer-assessed physical activity: The role of sociodemographic factors. Am J Epidemiol. 2014;179(6):781–790. 24500862 10.1093/aje/kwt330 PMC3939851

[50] Cassidy S, Fuller H, Chau J, Catt M, Bauman A, Trenell MI. Accelerometer-derived physical activity in those with cardio-metabolic disease compared to healthy adults: A UK biobank study of 52,556 participants. Acta Diabetol. 2018;55(9):975–979. 29808390 10.1007/s00592-018-1161-8 PMC6096713

[51] Hildebrand M, Van Hees VT, Hansen BH, Ekelund U. Age-group comparability of raw accelerometer output from wrist-and hip-worn monitors. Med Sci Sports Exerc. 2014;46(9):1816–1824. 24887173 10.1249/MSS.0000000000000289

[52] van Hees VT, Sabia S, Anderson KN, et al A novel, open access method to assess sleep duration using a wrist-worn accelerometer. PLoS One. 2015;10(11):e0142533. 26569414 10.1371/journal.pone.0142533 PMC4646630

[53] Rowlands AV, Maylor B, Dawkins NP, et al Stepping up with GGIR: Validity of step cadence derived from wrist-worn research-grade accelerometers using the verisense step count algorithm. J Sports Sci. 2022;40(19):2182–2190. 36384415 10.1080/02640414.2022.2147134

[54] Barry E, Galvin R, Keogh C, Horgan F, Fahey T. Is the timed up and go test a useful predictor of risk of falls in community dwelling older adults: A systematic review and meta- analysis. BMC Geriatr. 2014;14(1):14. 24484314 10.1186/1471-2318-14-14 PMC3924230

[55] Pickett SJ, Grady JP, Ng YS, et al Phenotypic heterogeneity in m.3243A>G mitochondrial disease: The role of nuclear factors. Ann Clin Transl Neurol. 2018;5(3):333–345. 29560378 10.1002/acn3.532 PMC5846390

[56] Grady JP, Pickett SJ, Ng YS, et al mtDNA heteroplasmy level and copy number indicate disease burden in m.3243A>G mitochondrial disease. EMBO Mol Med. 2018;10(6):e8262. 29735722 10.15252/emmm.201708262 PMC5991564

[57] de Laat P, Rodenburg RR, Roeleveld N, Koene S, Smeitink JA, Janssen MC. Six-year prospective follow-up study in 151 carriers of the mitochondrial DNA 3243 A>G variant. J Med Genet. 2021;58(1):48–55. 32439810 10.1136/jmedgenet-2019-106800

[58] Scholle LM, Zierz S, Mawrin C, Wickenhauser C, Urban DL. Heteroplasmy and copy number in the common m.3243A>G mutation-A post-mortem genotype-phenotype analysis. Genes (Basel). 2020;11(2):212. 32085658 10.3390/genes11020212 PMC7073558

[59] Cox BC, Pearson JY, Mandrekar J, Gavrilova RH. The clinical spectrum of MELAS and associated disorders across ages: A retrospective cohort study. Front Neurol. 2023;14:1298569. 38156086 10.3389/fneur.2023.1298569 PMC10753009

[60] Ng YS, Lax NZ, Blain AP, et al Forecasting stroke-like episodes and outcomes in mitochondrial disease. Brain. 2022;145(2):542–554. 34927673 10.1093/brain/awab353 PMC9014738

[61] Khasminsky V, Auriel E, Luckman J, et al Clinicoradiologic criteria for the diagnosis of stroke-like episodes in MELAS. Neurol Genet. 2023;9(4):e200082. 37426458 10.1212/NXG.0000000000200082 PMC10323819

[62] Jeppesen TD, Schwartz M, Olsen DB, Vissing J. Oxidative capacity correlates with muscle mutation load in mitochondrial myopathy. Ann Neurol. 2003;54(1):86–92. 12838523 10.1002/ana.10594

[63] Jeppesen TD, Schwartz M, Frederiksen AL, Wibrand F, Olsen DB, Vissing J. Muscle phenotype and mutation load in 51 persons with the 3243A>G mitochondrial DNA mutation. Arch Neurol. 2006;63(12):1701–1706. 17172609 10.1001/archneur.63.12.1701

[64] Parikh S, Karaa A, Goldstein A, et al Solid organ transplantation in primary mitochondrial disease: Proceed with caution. Mol Genet Metab. 2016;118(3):178–184. 27312126 10.1016/j.ymgme.2016.04.009

[65] Landfeldt E, Lindgren P, Bell CF, et al Health-related quality of life in patients with Duchenne muscular dystrophy: A multinational, cross-sectional study. Dev Med Child Neurol. 2016;58(5):508–515. 26483095 10.1111/dmcn.12938 PMC4949722

[66] Albrecht GL, Devlieger PJ. The disability paradox: High quality of life against all odds. Soc Sci Med. 1999;48(8):977–988. 10390038 10.1016/s0277-9536(98)00411-0

[67] Carlozzi NE, Schilling S, Kratz AL, Paulsen JS, Frank S, Stout JC. Understanding patient-reported outcome measures in Huntington disease: At what point is cognitive impairment related to poor measurement reliability? Qual Life Res. 2018;27(10):2541–2555. 29909483 10.1007/s11136-018-1912-6 PMC6295362

[68] Lee J, Zhang X. Is there really a proportional relationship between VO2max and body weight? A review article. PLoS One. 2021;16(12):e0261519. 34932594 10.1371/journal.pone.0261519 PMC8691647

[69] Krachler B, Savonen K, Komulainen P, Hassinen M, Lakka TA, Rauramaa R. Cardiopulmonary fitness is a function of lean mass, not total body weight: The DR’s EXTRA study. Eur J Prev Cardiol. 2020;22(9):1171–1179. 10.1177/2047487314557962 25381337

[70] Kaminsky LA, Imboden MT, Arena R, Myers J. Reference standards for cardiorespiratory fitness measured with cardiopulmonary exercise testing using cycle ergometry: Data from the fitness registry and the importance of exercise national database (FRIEND) registry. Mayo Clin Proc. 2017;92(2):228–233. 27938891 10.1016/j.mayocp.2016.10.003

[71] Taivassalo T, Abbott A, Wyrick P, Haller RG. Venous oxygen levels during aerobic forearm exercise: An index of impaired oxidative metabolism in mitochondrial myopathy. Ann Neurol. 2002;51(1):38–44. 11782982 10.1002/ana.10027

[72] Jeppesen TD, Olsen D, Vissing J. Cycle ergometry is not a sensitive diagnostic test for mitochondrial myopathy. J Neurol. 2003;250(3):293–299. 12638019 10.1007/s00415-003-0993-4

[73] Dandurand RJ, Matthews PM, Arnold DL, Eidelman DH. Mitochondrial disease. Pulmonary function, exercise performance, and blood lactate levels. Chest. 1995;108(1):182–189. 7606956 10.1378/chest.108.1.182

[74] Taivassalo T, Shoubridge EA, Chen J, et al Aerobic conditioning in patients with mitochondrial myopathies: Physiological, biochemical, and genetic effects. Ann Neurol. 2001;50(2):133–141. 11506394 10.1002/ana.1050

[75] Bogaard JM, Busch HF, Scholte HR, Stam H, Versprille A. Exercise responses in patients with an enzyme deficiency in the mitochondrial respiratory chain. Eur Respir J. 1988;1(5):445–452. 3139446

[76] McCoy J, Bates M, Eggett C, et al Pathophysiology of exercise intolerance in chronic diseases: The role of diminished cardiac performance in mitochondrial and heart failure patients. Open Heart. 2017;4(2):e000632. 28878952 10.1136/openhrt-2017-000632 PMC5574430

[77] Prince SA, Adamo KB, Hamel ME, Hardt J, Connor Gorber S, Tremblay M. A comparison of direct versus self-report measures for assessing physical activity in adults: A systematic review. Int J Behav Nutr Phys Act. 2008;5:56. 18990237 10.1186/1479-5868-5-56 PMC2588639

[78] Osanai S, Takahashi T, Enomoto H, et al Hypoxic ventilatory depression in a patient with mitochondrial myopathy, encephalopathy, lactic acidosis and stroke-like episodes. Respirology. 2001;6(2):163–166. 11422897 10.1046/j.1440-1843.2001.00318.x

[79] Suzukia Y, Taniyama M, Hata T, Miyaoka H, Atsumi Y, Matsuoka K. Sleep-wake dysrhythm in mitochondrial diabetes mellitus. Diabetes Res Clin Pract. 1997;35(1):61–62. 9113477 10.1016/s0168-8227(96)01358-7

[80] Flickinger J, Fan J, Wellik A, et al Development of a mitochondrial myopathy-composite assessment tool. JCSM Clin Rep. 2021;6(4):109–127. 35071983 PMC8782422

[81] European Medicines Agency (EMA). Guideline on clinical trials in small populations. (Adopted Reference Number: CHMP/EWP/83561/2005). 2006.

