# Supplementary material for: Outcomes misaligned in mitochondrial encephalomyopathy, lactic acidosis and stroke-like episodes (MELAS): implications for trial design
Source: Brain Commun. 2025 Sep 9;7(5):fcaf342. doi: 10.1093/braincomms/fcaf342 (PMC12495408; doi:10.1093/braincomms/fcaf342)
Supplement: fcaf342_Supplementary_Data [file fcaf342_supplementary_data.pdf]

Supplementary A. Summary of Registered Clinical Trials in Primary Mitochondrial Disease (as of 11 April 2025)

Trial Name	Investigational Product(s)	Study Design	Population	Status	Findings	Primary Outcome(s)
AIMM:<br>Randomised, double-blinded, placebo-controlled, adaptive design trial of the efficacy of acipimox in patients with Mitochondrial Myopathy<br>ISRCTN12895613<br>2018-002721-29	Acipimox	Phase Ia/Ib, randomised, DB, placebo-controlled using an adaptive design	≥16y, genetically confirmed PMM	Completed	Results pending publication	ATP content in skeletal muscle
MASS: MNGIE Allogeneic Hematopoietic Stem Cell Transplant Safety Study NCT02427178	AHSCT (allogeneic hematopoetic stem cell transplantation)	Phase I	5-55y, homozygous or compound heterozygous mutations in *TYMP*	Withdrawn, poor enrolment	Results NA	Engraftment success measured by neutrophil count (cells/L)
MABS01: MABs Therapy m.3243A>G Mutation Carriers NCT05063721	Autologous mesoangioblasts (MABs)	Open label	≥18, m.3243A>G mutation	Completed	PMID: 40682269<br>No serious AEs. Biopsies of treated muscle suggested mesoangioblast migration and early signs of regeneration.	AEs, blood flow (digital subtraction angiography) in lower leg, inflammation markers in blood and muscle
A study of Bezafibrate in Mitochondrial Myopathy NCT02398201	Bezafibrate	Open label	18-64y, m.3243A>G, PMM	Completed	PMID: 32107855<br>Reduction in complex IV-deficient muscle fibers ($p = 0.048$)	Respiratory chain enzyme activity
MOUNTAINSIDE: A Study to Evaluate ASP0367 in Participants With PMM NCT04641962	Bocidelpar/ASP0367/MA-0211	Phase 2, randomized, DB, placebo-controlled	18-65y, diagnosis of PMM (molecular mtDNA or nDNA genetic abnormality), symptoms or physical examination myopathy findings that are the predominant symptoms of their mitochondrial disorder	Terminate due to failure to meet the pre-specified criteria for efficacy	Results NA	Safety and tolerability (nature, frequency ,severity of TEAEs), body weight, ECG, laboratory parameters, vital signs; suicidal ideation and/ or behaviour as assessed C-SSRS; distance waked in 6MWT
Phase III Trial of Coenzyme Q10 in Mitochondrial Disease NCT00432744	Coenzyme Q_{10}	Phase 3, randomised, crossover. Triple masking (Participant, Care Provider, Investigator)	12m-17y, biochemical proof of a deficiency of complex I, III or IV or molecular genetic proof of a mutation in mtDNA, or an nDNA mutation in a gene known to be associated with dysfunction of the electron transport chain (e.g., SURF1)	Completed	No significant difference in primary outcomes	GMFM 88);<br>The Pediatric Quality of Life Scale
RP103-MITO-001:<br>Open-Label, Dose-Escalating Study Assessing Safety, Tolerability, Efficacy, of RP103 in Mitochondrial Disease<br>NCT02023866	Cysteamine bitartrate/ RP103	Open label, dose-escalating	≥6 and <18y, genetically confirmed known mutation. Diagnosis of inherited mitochondrial disease other than Friedreich's ataxia (FRDA)	Completed	Results NA	NPMDS score sections I-IV
RP103-MITO-002:<br>A Long-term Extension of Study to Assess Cysteamine Bitartrate Delayed-release Capsules (RP103) in Children With Inherited Mitochondrial Disease<br>NCT02473445	Cysteamine bitartrate/RP103	Phase 2, open label extension	Completed all visits in RP103-MITO-00	Terminated. Sponsor ended development of due to lack of efficacy in base study RP103-MITO-001.	RP103 may reduce oxidative stress	NPMDS score sections I-IV
TK0102: An Open-Label Study of Continuation Treatment With Combination Pyrimidine Nucleosides in Patients With TK2 Deficiency (Continuation) NCT03845712	Deoxycytidine (dC) and deoxythymidine (dT)/MT1621	Phase 2, open label	Confirmed genetic mutation in the *TK2* gene	Active, not recruiting	Results NA	Safety as AEs, laboratory measurements, ECG

Trial Name	Investigational Product(s)	Study Design	Population	Status	Findings	Primary Outcome(s)
MT-1621-104: A Study of the Efficacy and Safety of MT1621 in Thymidine Kinase 2 (TK2) Deficiency (Treatment naïve) NCT04581733	Deoxycytidine (dC) and deoxythymidine (dT)/MT1621	Phase 3b, open label	<18y, diagnosis of *TK2* deficiency based on confirmed disease-causing mutation(s) in the *TK2* gene	Withdrawn. Sponsor decision	Results NA	Proportion of subjects acquiring a motor milestone
dC-dT-MDS: Deoxynucleosides Pyrimidines as Treatment for Mitochondrial Depletion Syndrome NCT04802707	Deoxycytidine (dC) and deoxythymidine (dT	Phase 2, open label,	0-60y, diagnosis of a mitochondrial depletion disorder, pathogenic variant(s) in one of the following genes: *POLG*, *C10orf2*, *RRM2B*, *MPV17*, *SUCLA2*, *SUCLG1*, *FBXL4*	Recruiting	Results NA	Responder rate vs. non-responder rate - defined as having ≥2 of (1) EEG improvement, (2) decreased seizure frequency, (3) cognitive improvement, (4) caregiver impression of improvement, (5) clinical improvement, (6) Normal organics and metabolism functions
TEETPIM: Trial of Erythrocyte Encapsulated Thymidine Phosphorylase In Mitochondrial Neurogastrointestinal Encephalomyopathy NCT03866954	EE-TP	Phase 2, open label, multiple dose	≥12y, diagnosed with MNGIE	Withdrawn. Change of circumstances with commercial partner	Results NA	Safety as TEAEs, laboratory measurements, vital signs, ECG, BMI, use of concomitant medication(s), pharmacodynamics
MMPOWER; SPIMM-201: Safety, Tolerability, and Efficacy of MTP-131 for the Treatment of Mitochondrial Myopathy NCT02367014	Elamipretide/MTP-131	Phase 1/2, randomized, DB, placebo-controlled multiple ascending dose	Genetically confirmed PMM	Completed	PMID: 29500292 Dose-dependent increase in 6MWT distance walked (p = 0.014); Highest dose increase walk distance vs. placebo (p = 0.053)	Distance walked in 6MWT
MMPOWER-2; SPIMM-202: Safety, Tolerability, Efficacy of MTP-131 for Treatment of Mitochondrial Disease in Subjects From the MMPOWER Study NCT02805790	Elamipretide/MTP-131	Phase 2, randomized, DB, placebo-controlled, crossover	Completed participation in the SPIMM-201	Completed	PMID: 32096613 Clinically meaningful increase in 6MWT did not achieve statistical significance.	Distance walked in 6MWT
SPIMM-203: Open-Label Extension Trial to Characterize the Long-term Safety and Tolerability of Elamipretide in Subjects With Genetically Confirmed PMM NCT02976038	Elamipretide/MTP-131	Phase 2, open label extension study	Completed the End of Study Visit in SPIMM-202	Terminated. Registration trial did not meet the primary end points	Results pending publication	Distance walked in 6MWT
MMPOWER-3; SPIMM-301: A Trial to Evaluate Safety and Efficacy of Elamipretide PMM Followed by Open-Label Extension NCT03323749	Elamipretide/MTP-131	Phase 3, randomized, DB, parallel-group, placebo-controlled	≥16 and ≤ 80y, genetically confirmed PMM	Terminated. Part1, DB portion of the trial did not meet the primary end points	PMID: 37268435 Primary endpoints not met	Distance walked in 6MWT; PMMSA total fatigue score
NuPower; SPIMD-301: Study to Evaluate Efficacy and Safety of Elamipretide in Subjects With Primary Mitochondrial Disease From Nuclear DNA Mutations (nPMD) NCT05162768	Elamipretide/MTP-131	Phase 3, randomized, DB, parallel-group, placebo-controlled	≥18 and ≤70y, nPMD, myopathy	Completed	Results pending publication	Distance walked in 6MWT

Trial Name	Investigational Product(s)	Study Design	Population	Status	Findings	Primary Outcome(s)
TAZPOWER:<br>A Trial to Evaluate Safety, Tolerability and Efficacy of Elamipretide in Subjects With Barth Syndrome<br>NCT03098797	Elamipretide/MTP-131	Phase 2 randomized, DB, placebo-controlled crossover	≥12y, genetically confirmed Barth Syndrome	Completed	PMID: 38602181 6MWT improvements (cumulative, p = .003).<br>BTHS-SA total fatigue scores (p = 0.21)	Distance walked in 6MWT;<br>BTHS-SA total fatigue score
SPIES-006; SPIES-007:<br>An Intermediate Size Expanded Access Protocol of Elamipretide<br>NCT04689360	Elamipretide/MTP-131	Expanded Access	≥1 and ≤ 80y or ≥12y for Barth Syndrome in SPIES-007.<br>Genetically confirmed PMD including Barth Syndrome	Available	Results NA	NA
SPILH-201:<br>A Study Investigating the Safety, Tolerability, and Efficacy of Elamipretide Topical Ophthalmic Solution for Treatment of LHON<br>NCT02693119	Elamipretide/MTP-131	Phase 2, prospective, randomized, double-masked, vehicle-controlled, plus open label extension	≥18 and ≤ 50y at the time of loss of vision in the second eye.<br>LHON, genetic mtDNA mutation m.11778G>A	Completed	PMID: 37923251<br>Well tolerated, majority of AEs were mild to moderate and resolved spontaneously.	Incidence and severity of ocular TEAEs.
LHON-Plus: A Basket Clinical Study to Assess Glycerol Tributyrate in Patients With MELA) or LHON-Plus<br>NCT06792500	Glycerol tributyrate	Phase 1, parallel arm non-randomized dose-escalation, open label basket	18-65y, confirmed molecular diagnosis of MELAS or LHON-Plus, symptomatic. m.3243A>G (MELAS), m.11778G>A (LHON), or<br>or a mitochondrial pathogenic variant solely mapping in a mitochondrial gene encoding a mitochondrial subunit of Complex I	Not yet recruiting	Results NA	Dose safety (TEAEs), potential efficacy on bioenergetic parameters for the oxidative phosphorylation pathway and/or mitochondrial ATP rate production
Clinical Study of Hydroxytyrosol (HT) in Mitochondrial Diseases<br>NCT04543968	Hydroxytyrosol	Open label	3-18y, confirmed to have pathogenic disease-associated nDNA or mtDNA mutations.	Completed	Results NA	International Paediatric Mitochondrial Disease Score
SNT-II-003; RHODOS:<br>Study to Assess Efficacy,Safety and Tolerability of Idebenone in the Treatment of LHON NCT00747487	Idebenone/ Raxone	Phase 2, DB, randomized, placebo-controlled	≥14 and < 65y, impaired visual acuity in at least one eye due to LHON (m.3460G>A, m.11778G>A, m.14484T>C mutations)	Completed	PMID: 21788663<br>Primary endpoint did not reach statistical significance. Secondary endpoint improvement vs. placebo group in patients with discordant visual acuities	Best recovery of logMAR visual acuity in either right or left eye
SNT-II-007: Study of Idebenone in the Treatment of MELAS<br>NCT00887562	Idebenone/Raxone	Phase 2a, DB, randomized, placebo-controlled, dose-finding	8-65y, MELAS with confirmed A3243G, or evidence of CNS involvement.	Completed	No significant difference in primary outcomes	Cerebral lactate concentration as measured by MRS
SNT-IV-003; PAROS:<br>Post Authorisation Safety Study With Raxone in LHON Patients<br>NCT02771379	Idebenone/Raxone	Prospective	Prescribed Raxone® for the treatment of LHON	Completed	Results NA	Long-term safety profile assessed by incidence of AEs
SNT-IV-005; LEROS:<br>Study to Assess the Efficacy and Safety of Raxone in LHON Patients<br>NCT02774005	Idebenone/Raxone	Phase IV, open label, natural history-controlled	≥12, impaired visual acuity in affected eyes due to LHON (up to 5 years after symptom onset)	Completed	PMID: 38428428<br>Primary endpoint met, confirms long-term efficacy	Proportion of eyes with clinically relevant recovery of visual acuity
KL1333 2018-102:<br>A Phase Ia/Ib, SAD and MAD Study of KL1333 in Healthy Subjects and Patients With PMD<br>NCT03888716	KL1333	Phase 1a/b, DB, randomized, placebo-controlled, single and multiple oral dose	18-75y, genetically confirmed PMD, clinically stable, BMI 15.0–32.0 kg/m^2	Completed	PMID: 39657714<br>Well tolerated, with dose-dependent gastrointestinal side effects. Informed Phase 2 studies	Safety (incidence/severity of AEs, laboratory analytes, ECG, vital signs, physical examination)
Therapeutic regimen of l-arginine for MELAS: 9-year, prospective, multicenter, clinical research	L-arginine (oral and intravenous (IV)	OL-MELAS Research: Integrated pooled data from two, 2-year, phase 3, prospective, open label	Oral: Developed stroke-like episodes in the last 2y, m.3243A>G, never been treated with oral l-arginine, and clinical	Completed	PMID: 30269300<br>Oral: Extended the interictal phase (*p* = 0.0625) and decreased the incidence and severity of ictuses.	Oral: MELAS stroke scale<br><br>IV: Improvement rates of headache and nausea/ vomiting at 2 h after

Trial Name	Investigational Product(s)	Study Design	Population	Status	Findings	Primary Outcome(s)
Center for Clinical Trials, Japan Medical Association: MACTR-IIA00023 and JMACTR-IIA00025		trials of oral and IV l-arginine	manifestations of stroke-like episodes were evaluable.<br><br>IV: Diagnosed with MELAS, m.3243A>G, and developed an ictus of stroke-like episodes within the previous 6 h		IV: Improved the rates of symptoms: headache, nausea/vomiting, impaired consciousness, and visual disturbance	completion of the initial IV administration
The Effect of Arginine and Citrulline Supplementation on Endothelial Dysfunction in Mitochondrial Diseases NCT02809170	L-arginine,<br>L-Citrulline	Open label, randomised crossover	3-18y, confirmed diagnosis of mitochondrial disease with multi-organ disease involving neurological and/or muscular systems	Completed	Reactive hyperemic index increased	Reactive hyperemic index to reflects endothelial function, measured using the EndoPAT
L-Citrulline Dose Finding Safety Study in MELAS NCT03952234	L- citrulline	Phase 1, open label, dose finding	18-65y, clinical diagnosis of MELAS, m.3243A>G	Completed	Results NA	Incidence of dose limiting toxicities to establish maximum tolerated dose
GS-LHON/CLIN/01: Safety Evaluation of Gene Therapy in LHON Patients NCT02064569	Lenadogene nolparvovec (GS010)	Phase 1/2, open label, dose escalation	≥18, genetic confirmation of m.11778G>A *ND4* LHON, visual acuity ≤ 1/10 of the less functional eye	Completed	PMID: 29426586<br>Well tolerated. 9E10 vg/eye dose defined as the best benefit/risk ratio; chosen for the ensuing studies.	Incidence of local and general AEs and SAEs
GS-LHON-CLIN-03A; RESCUE: Efficacy Study of GS010 for the Treatment of Vision Loss up to 6 Months From Onset in LHON Due to the ND4 Mutation NCT02652767	Lenadogene nolparvovec (GS010)	Phase 3, randomized, DB, sham-controlled	≥15, LHON patients with vision loss ≤6 months in the first-affected eye	Completed	PMID: 34108929<br>Primary outcome not met	Visual acuity derived from the ETDRS chart
GS-LHON-CLIN-03B; REVERSE: Efficacy Study of GS010 for Treatment of Vision Loss From 7 Months to 1 Year From Onset in LHON Due to the ND4 Mutation NCT02652780	Lenadogene nolparvovec (GS010)	Phase 3, randomized, DB, sham-controlled	≥15, LHON patients with vision loss between 6 months to 1y in both eyes	Completed	PMID: 34108929<br>Primary end point was not met (p = 0.894)	ETDRS Visual Acuity (Quantitative Score)
GS-LHON-CLIN-05; REFLECT: Efficacy & Safety Study of Bilateral IVT Injection of GS010 in LHON Subjects Due to the ND4 Mutation for up to 1 Year NCT03293524	Lenadogene nolparvovec (GS010)	Phase 3, randomized, DB, sham-controlled	≥15, clinically manifested vision loss due to m.11778G>A *ND4* LHON, to any extent, in at least one eye.	Unknown	Results reported in GenSight Biologics Financial press release (12/02/2025): Sustained efficacy at 5y (75% had ≥15-letter gain from nadir). Favourable safety.	BCVA reported using Log of the Minimal Angle of Resolution (LogMAR)
RESTORE: RESCUE and REVERSE Long-term Follow-up NCT03406104	Lenadogene nolparvovec (GS010)	Phase 3, prospective long-term follow-up	Treated with GS010 IVT injection in either RESCUE or REVERSE	Completed	PMID: 34108929	No. of eyes with ocular AEs, from 2-5y post-treatment
REN001-101:<br>A Study of the Safety of REN001 in Patients With PMM NCT03862846	Mavodelpar/REN001	Phase 1, open label	≥16y, genetically confirmed PMM	Terminated. COVID-19 sufficient data gathered to achieve the study objective	Results pending publication	AEs as a measure of safety and tolerability
REN001-201; STRIDE:<br>An Efficacy and Safety Study of 24 Week Treatment With Mavodelpar (REN001) in PMM Patients NCT04535609	Mavodelpar/REN001	Randomized, DB, placebo-controlled, parallel group	≥18y, genetically confirmed PMM	Completed	Results pending publication	Distance walked in 12MWT
Stride Ahead: REN001-202:<br>An Open Label, Long Term Safety Study of REN001 in PMM Patients NCT05267574	Mavodelpar/ REN001	Open label	Completed REN001-201 or participated in REN001-101 (mtDNA-PMM) or nDNA-PMM	Terminated. Parent study failed to show therapeutic effect	Results NA	No. of participants with AEs and severity
Study on the Effects of TTI-0102 for Patients with MELAS Syndrome 2023-506723-28-00	Mercaptamine-Pantetheine Disulfide Acetate /TTI-0102	Phase 2, randomized, DB, placebo-controlled	6-60 y, confirmed diagnosis of MELAS with specific genetic mutations			12MWT distance; Fatigue Severity Scale; Quality of Life questionnaire (WHOQOL-BREF) (Primary is not specified)

Trial Name	Investigational Product(s)	Study Design	Population	Status	Findings	Primary Outcome(s)
PLC-PMD-01-IL: A First in Human Study to Evaluate the Safety of Infusion of MNV-BM-PLC (Autologous CD34+ Cells Enriched With Placenta Derived Allogeneic Mitochondria) in Patients With PPMD Associated With mtDNA Mutation or Deletion NCT04548843	MNV-BM-PLC (Autologous CD34+ Cells Enriched With Placenta Derived Allogeneic Mitochondria)	Phase I, open label, dose-escalation. Sequential assignment	4-18y, molecular diagnosis of PMD	Unknown	Results NA	No. of participants with TTEAEs assessed by CTCAE v5.0; hemoglobin; bsolute neutrophil count; platelet count
Low Residue Diet Study in Mitochondrial Disease) NCT03388528	Low residue diet	Phase 2, Open label	≥18y, genetic or biochemical confirmation of mitochondrial disease	Completed	PMID: 39132075 Well tolerated with no AEs. No change in stool frequency	Tolerability (via food diaries); stool consistency (Bristol Stool Form scale)
MNV-BM-BLD-001-IL: A Study to Evaluate the Safety and Therapeutic Effects of Transplantation of MNV-BM-BLD in Pediatric Patients With Pearson Syndrome NCT03384420	MNV-BM-PLC	Phase I/2, open label, single Dose	3-18y, diagnosed with Pearson Syndrome, as verified by molecular identification of a defect in the mitochondrial DNA	Completed	Results NA	No. of participants with TTEAEs; IPMDS (International Pediatric Mitochondrial Disease Scale)
LS007. ABI-009 (Nab-sirolimus) in Patients With Genetically-confirmed Leigh or Leigh-like Syndrome NCT03747328	Nab-sirolimus/ABI-009	Phase 2a, open-label	2-17y, genetically-confirmed Leigh or Leigh-like syndrome, and clinical evidence	Withdrawn	Results NA	Incidence of TEAEs
Study of N-acetylcysteine in the Treatment of Patients With the m.3243A>G Mutation and Low Brain Glutathione Levels NCT05241262	N-acetylcysteine (NAC)	Open label	8-80y, low brain glutathione (GSH) levels determined by MRSI, genetic confirmation of m.3243A>G	Recruiting	Results NA	Maximum tolerated dose of NAC
NiaMIT; NiaMIT_0001: Niacin Supplementation in Healthy Controls and Mitochondrial Myopathy Patients NCT03973203	Niacin	Open label	17-80, pure mitochondrial myopathy, with no major other symptoms or manifestations, caused by single or multiple deletions of mtDNA	Completed	PMID: 32386566 Increased blood and muscle NAD+ of patients to the level of their controls. Muscle strength and mitochondrial biogenesis increased in all subjects. Muscle metabolome shifted toward controls and liver fat decreased	NAD+ and related metabolite levels in blood and muscle
NiaMIT Continuation With Early-stage Mitochondrial Myopathy Patients NCT04538521	Niacin	Open label	≥17, early-stage, genetically diagnosed PMM, with no major other symptoms or manifestations, caused by single or multiple deletions of mtDNA	Completed	Results NA	NAD+ and related metabolite levels in blood and muscle
A Study to Evaluate Vitamin B3 Derivative to Treat Mitochondrial Myopathy NCT05590468	Nicotinamide riboside	Phase 2, randomized, DB, placebo-controlled	>18y, Biochemically and/or genetically confirmed PMM	Recruiting	Results NA	Distance walked in 6MWT
Nicotinamide Riboside and Mitochondrial Biogenesis NCT03432871	Nicotinamide riboside	Open label	18-70y, PEO, caused by a single deletion of mtDNA or mitochondrial disease caused by m.3243A>G or A>T mutation	Completed	Results NA	Bioavailability; safety (AEs, blood analytes, vital signs); mitochondrial biogenesis (31P-MRS, respiratory chain enzyme analysis and mtDNA quantification)
MOTOR, RTA 408-C-1403: RTA 408 Capsules in Patients With Mitochondrial Myopathy NCT02255422	Omaveloxolone/RTA 408	Phase 2, randomized, DB, placebo-controlled, sequential assignment, dose-escalation	≥18 and ≤75y, genetically confirmed PMM	Completed	PMID: 31896620 No change in primary outcome vs. placebo. Highest dose reduced HR and lactate during submaximal exercise (exploratory endpoint)	Peak workload (in Watts/kg) during exercise test

Trial Name	Investigational Product(s)	Study Design	Population	Status	Findings	Primary Outcome(s)
PMD-OPTION: OMT-28 in Patients With PMD NCT05972954	OMT-28	Phase 2a, open label	18-60y, mutation resulting in PMD, including m3243A>G, m8344A>G, and single mtDNA deletions. Diagnosis of cardiomyopathy as defined, myopathy	Active, not recruiting	Results NA	No. of TEAEs; No. patients with a between phase difference in GDF-15 of ≥20% decrease
Efficacy Study of Gene Therapy for The Treatment of Acute LHON Onset Within Three Months NCT03428178	rAAV2-*ND4*	Open label	8-60y, genetic confirmation of m.11778G>A *ND4* LHON	Unknown	PMID: 30878986	BCVA
RAVCT-2. Safety and Efficacy Study of rAAV2-ND4 Treatment of Leber Hereditary Optic Neuropathy (LHON) (rAAV2-ND4) NCT01267422	rAAV2-*ND4*	Open label	8-60y, genetic confirmation of m.11778G>A *ND4* LHON	Completed	PMID: 30878986<br>4/9 patients showed a clinically meaningful improvement of BCVA in both eyes.	BCVA;<br>CD3+/CD4+/CD8+ test
A Single Intravitreal Injection of rAAV2-ND4 for the Treatment of LHON NCT03153293	rAAV2-*ND4*	Phase 2/3, open label	10-65y, LHON and m.11778G>A	Unknown	PMID: 32096343<br>Rapid and significant BCVA improvement reported within 3 days in at least 1 eye of 36.2% of patients and in both eyes in 11.4% of patients	BCVA, computerized visual field
Resveratrol Supplementation in Patients With Mitochondrial Myopathies and Skeletal Muscle Fatty Acid Oxidation Disorders NCT03728777	Resveratrol	DB, placebo-controlled, cross over	≥18 and ≤80y, genetically verified mitochondrial disorder or a fatty acid oxidation deficiency	Completed	Results NA	Decrease in HR during constant load cycling exercise
RG2133 (2',3',5'-Tri-O-Acetyluridine) in Mitochondrial Disease NCT00060515	RG2133 (2',3',5'-tri-O-acetyluridine)	Phase 1, dose-escalation	≥3y, mitochondrial disease	Terminated	Results NA	NA
Safety Study of an Adeno-associated Virus Vector for Gene Therapy of LHON NCT02161380	scAAV2-P1ND4v2 (AAV-*ND4*)	Phase 1, open label, dose-escalation	≥15y, LHON and m.11778G>A	Active, not recruiting	PMID: 28647203<br>No dose response was observed. Bilateral BCVA improvement reported in 4 /6 patients who showed signs of efficacy	No. of treatment related AEs
KH176-201; The KHENERGY Study NCT02909400	Sonlicromanol/ KH176	Phase 2, DB, randomized, placebo-controlled, two-way cross-over	≥18y, m.3243A>G mutation and clinical signs of mitochondrial disease	Completed	PMID: 30058726<br>No improvement in gait parameters. positive effect on alertness and mood (secondary outcome)	Motor abnormalities and movement characteristics
KH-176-202: The KHENERGYZE Study NCT04165239	Sonlicromanol/ KH176	Phase 2b, DB, open-label, randomised, placebo-controlled, three-way cross-over	≥18y. Confirmed m.3243A>G mutation and clinical signs of mitochondrial disease	Completed	Results NA	Attention domain score of cognitive functioning, (visual Identification Test of the Cogstate computerised cognitive testing battery)
KH176-203; The KHENEREXT Study NCT04604548	Sonlicromanol/ KH176	Phase 2, open label	Completed KH176-202	Completed	Results NA	Frequency of TEAEs
KH176-204; The KHENERGYC Study NCT04846036	Sonlicromanol/ KH176	Phase 2, randomized, DB, placebo controlled, parallel-group	0m-17y, genetically confirmed PMD	Suspended. Strategic company decision - not related to safety concerns		Motor Symptom Severity as assessed with the GMFM-88
KH176-301; KHENERFIN: A Trial to Evaluate the Efficacy and Safety of Sonlicromanol in PMD NCT06451757	Sonlicromanol/ KH176	Phase 3, randomised, DB, placebo-controlled, parallel-group	≥18y, confirmed m.3243A>G	Not yet recruiting		Neuro-QoL Fatigue SF v1; time to complete 5XSST
AAAQ7552: Treatment of TK2 Deficiency with Thymidine and Deoxycytidine NCT03639701	Thymidine	Phase ½, open label	Genetically confirmed diagnosis of *TK2* deficiency, symptomatic	Active, not recruiting	Results NA	Blood analysis (ALT, AST, GGT, lymphocyte count, creatinine), ECG, PROMIS scale v1.0 -

Trial Name	Investigational Product(s)	Study Design	Population	Status	Findings	Primary Outcome(s)
						Gastrointestinal Diarrhea 6a score
EPI743-12-002: Safety and Efficacy Study of EPI-743 in Children With Leigh Syndrome NCT01721733	Vatiquinone/ EPI-743	Phase 2B, randomized, DB, placebo controlled with an extension phase	<18y, clinical and MRI diagnosis of Leigh syndrome	Completed	Results NA	NPMDS sections 1–3 score
EPI-743 for Metabolism or Mitochondrial Disorders NCT01642056	Vatiquinone/ EPI-743	Cross over. Double masking (Participant, Investigator)	2-11y, disorders of impaired energy utilisation/oxidation-reduction	Completed	Results NA	NPMDS sections 1–3
EPI-2009-1: EPI-743 for Mitochondrial Respiratory Chain Diseases NCT01370447	Vatiquinone/ EPI-743	Emergency Use	Genetically confirmed mitochondrial respiratory chain disease, deemed to be within 90 days of end-of-life care	No longer available	Results NA	Incidence of AEs
EPI743-12-002: Safety and Efficacy Study of EPI-743 in Children With Leigh Syndrome NCT01721733	Vatiquinone/ EPI-743	Phase 2B, randomized, DB, placebo-controlled	<18y, clinical and MRI diagnosis of Leigh syndrome	Completed	Fewer patients requiring hospitalization or experiencing SAEs	NPMDS sections 1–3
EPI743-13-023 Long-Term Safety and Efficacy Evaluation of EPI-743 in Children With Leigh Syndrome NCT02352896	Vatiquinone/ EPI-743	Open label	Completed EPI743-12-002	Completed	Progressive decline in hospitalizations and SAEs	NPMDS sections 1–3; Dose-limiting SAEs
Phase 2 Study of EPI-743 in Children With Pearson Syndrome NCT02104336	Vatiquinone/ EPI-743	Phase 2 , open label	<18y, genetically confirmed Pearson syndrome	Terminated. Results from other studies did not support continuation	Results NA	Occurrence of episodes of sepsis, metabolic crisis or hepatic failure
PTC743-MIT-001-EP: A Study to Evaluate Efficacy and Safety of Vatiquinone for Treating Mitochondrial Disease in Participants With Refractory Epilepsy (MIT-E) NCT04378075	Vatiquinone/ EPI-743	Phase 2/3, parallel-arm, DB, placebo-controlled	Genetic confirmation of inherited mitochondrial disease with associated epilepsy phenotype	Terminated. Sponsor decision	Results NA	No. of observable motor seizures per 28 days
Phase 2a Study of IW-6463 in Adults Diagnosed With MELAS NCT04475549	Zagociguat/ IW-6463	Phase 2a, open label	≥18y, genetic confirmation of a known mitochondrial disease mutation, neurological features of MELAS	Terminated due to enrolment challenges.	Results NA	No. of participants with study drug dose reductions or discontinuations due to ≥1 TEAE. No. of participants with ≥1 AE, TEAE, SAE or AESI
PRIZM A Phase 2b Study of Zagociguat in Patients with MELAS NCT06402123	Zagociguat/ IW-6463	Phase 2b, randomized, DB, placebo-controlled crossover	18-75y, diagnosed with MELAS based on the presence of each of: documented pathogenic variant in a mtDNA gene, history of ≥ stroke-like episodes with MRI findings consistent with stroke-like lesions	Recruiting	Results NA	TEAEs incidence; PROMIS Fatigue MELAS SF scores; Groton Maze Learning scores; International digit symbol substitution test scores.

Abbreviations: 5XSST = Five Times Sit-To-Stand Test; 6MWT = six metre walk test; 12MWT = twelve metre walk test; AAV = Adeno-Associated Virus; AEs = adverse events; AESI = TEAE of Special Interest; ATP = adenosine triphosphate; BCVA = Best-Corrected Visual Acuity; BTHS-SA = BarTH Syndrome Symptom Assessment; CNS = central nervous system; C-SSRS = Columbia-Suicide Severity Rating Scale; DB = double-blind; EE-TP = Erythrocyte Encapsulated Thymidine Phosphorylase; ETDRS = Early Treatment Diabetic Retinopathy Study chart; GMFM-88 = Gross Motor Function Measure-88; HR = heart rate; intravenous = IV; LHON = Leber's Hereditary Optic Neuropathy; MELAS = Mitochondrial Encephalopathy Lactic Acidosis & Stroke-like Episodes; MRS = magnetic resonance spectroscopy; MRSI = magnetic resonance spectroscopic imaging; MNGIE = Mitochondrial neurogastrointestinal encephalomyopathy; MRI, magnetic resonance imaging; mtDNA = mitochondrial DNA; NA = not available; nPMD = nuclear DNA Primary Mitochondrial Disease; NPMDS = Newcastle Paediatric Mitochondrial Disease Scale; PEO = Progressive external ophthalmoplegia; PMM = Primary Mitochondrial Myopathy; PMMSA = Primary Mitochondrial Myopathy Symptom Assessment; PROMIS = Patient-Reported Outcome Measurement Information System; rAAV2-*ND4* = recombinant Adeno-Associated Virus-NADH dehydrogenase, subunit 4 (complex I); SAE = serious adverse events; SF = short form; TEAEs = Treatment emergent adverse events; TK2, thymidine kinase 2; y = years.

Supplementary B

Methods

Inclusion Criteria

Patients will only be eligible if they meet all of the following criteria.

1. Male and Females over the age of 18 years at the time of screening
2. Participants must harbour the m.3243A>G pathogenic variant disease (confirmed by assessment of heteroplasmy in blood and urine samples).
3. Participants with clinical features of mitochondrial disease, including those with stroke-like episode (classified as MELAS phenotype).
4. Participants who are currently asymptomatic but are deemed at risk of clinical manifestation due to their m.3243A>G variant heteroplasmy level.
5. Females of child-bearing age must have a negative serum/urine pregnancy test.
6. Capacity to provide informed consent taken before any study related activities.
7. Ability and willingness to adhere to the protocol, including all appointments.
8. Ability to read and converse in English.

Exclusion Criteria

Patient will not be eligible if they meet any of the following criteria

1. Previous history of contraindicated conditions including stroke, brain lesion(s) or tumour.
2. Positive serum/urine pregnancy test.
3. Abnormal ECG at screening.
4. Abnormal clinical results as determined by physician.
5. Patient without capacity to provide informed consent.
6. Patient's unwillingness to adhere to the protocol, including all appointments.
7. Language barriers preventing patients from reading and conversing in English

Results

Table S1 Frequency of clinical features

NMDAS item sub scores (≥ 2)	All m.3243A>G (*n* = 16)	MELAS syndrome (*n* = 6)	non-MELAS (*n* = 10)
**Gastro-intestinal symptoms**[a], No. (%)	14 (88%)	5 (83%)	9 (90%)
NMDAS sub score: 2		1	5
NMDAS sub score: 3		4	3
NMDAS sub score: 4		-	1
**Hearing loss**[b], No. (%)	10 (63%)	6 (100%)	4 (40%)
NMDAS sub score: 2		4	4
NMDAS sub score: 3		1	-
NMDAS sub score: 4		1	-
**Migraine**[c], No. (%)	10 (63%)	4 (67%)	6 (60%)
NMDAS sub score: 2		1	-
NMDAS sub score: 4		1	2
NMDAS sub score: 5		2	4
**Exercise intolerance**[d], No. (%)	9 (56%)	4 (67%)	5 (50%)
NMDAS sub score: 2		2	1
NMDAS sub score: 3		2	3
NMDAS sub score: 4		-	1
**Gait instability**[e], No. (%)	9 (56%)	5 (83%)	4 (40%)
NMDAS sub score: 2		4	2
NMDAS sub score: 3		1	2
**Psychiatric disturbance**[f], No. (%)	9 (56%)	4 (67%)	5 (45%)
NMDAS sub score: 2		2	2
NMDAS sub score: 3		2	3
**Myopathy**[g], No. (%)	8 (50%)	4 (67%)	4 (40%)
NMDAS sub score: 2		4	4
**Diabetes mellitus**[h], No. (%)	8 (50%)	3 (50%)	5 (45%)
NMDAS sub score: 2		1	-
NMDAS sub score: 4		1	2
NMDAS sub score: 5		1	3
**Cognitive impairment**[i], No. (%)	6 (38%)	5 (83%)	1 (10%)
NMDAS sub score: 2		1	-
NMDAS sub score: 3		2	1
NMDAS sub score: 4		2	-
**Seizures**[j], No. (%)	4 (25%)	4 (67%)	0
NMDAS sub score: 2		2	-
NMDAS sub score: 3		2	-
**Ataxia**[k], No. (%)	4 (25%)	3 (50%)	1 (10%)
NMDAS sub score: 2		2	1
NMDAS sub score: 3		1	-
**Respiratory muscle weakness**[l], No. (%)	4 (25%)	3 (50%)	1 (10%)
NMDAS sub score: 2		2	1
NMDAS sub score: 3		1	-
**Ptosis**[m], No. (%)	4 (25%)	2 (33%)	2 (20%)
NMDAS sub score: 3		2	2
**Cardiac**[n] No. (%)	3 (19%)	2 (33%)	1 (10%)
NMDAS sub score: 3		2	-
NMDAS sub score: 4		-	1

Data are frequency of clinical characteristics, based on the Newcastle Mitochondrial Disease Adult Scale (NMDAS) item sub-scores ≥ 2. Higher NMDAS score indicates greater disease seventy.

a. Gastro-intestinal symptoms: 2 = Occasional symptoms of 'irritable bowel' (pain, bloating or diarrhoea) with long spells of normality; 3 = Frequent symptoms most weeks or severe constipation with bowels open less than once/week or need for daily medications; 4 = Dysmotility requiring admission or persistent and/or recurrent anorexia/vomiting/weight loss.

b. Hearing loss (with or without hearing aids): 2 = Mild deafness. Missing words in presence of background noise, fully corrected with hearing aids; 3 = Moderate deafness. Regularly requiring repetition, not fully corrected with hearing aids; 4 = Severe deafness. Poor hearing even with hearing aids.
c. Migraine headaches: 2 = One day per month; 4 = Three days per month; 5 = ≥ Four days per month.
d. Exercise intolerance: 2 = Able to walk < 1000m on the flat. Restricted on inclines or stairs - rest needed after 1 flight (12 steps); 3 = Able to walk < 500m on the flat. Rest needed after 8 steps on stairs; 4 = Able to walk < 100m on the flat. Rest needed after 4 steps on stairs.
e. Gait stability: 2 = Gait reasonably steady. Aware of impaired balance. Occasionally off balance when walking; 3 = Unsteady gait. Always off balance when walking. Occasional falls. Gait steady with support of stick or person.
f. Psychiatric disturbance: 2 = Mild & persistent (lasting more than 3 months) or recurrent. Patient has consulted GP; 3 = Moderate & warranting specialist treatment (e.g. from a psychiatrist) - eg. bipolar disorder or depression with vegetative symptoms (insomnia, anorexia, abulia etc).
g. Myopathy: 2 = Mild but clear proximal weakness in hip flexion and shoulder abduction (MRC 4/5). Minimal weakness in elbow flexion and knee extension (MRC 4+/5- both with joint at 90 degrees).
h. Diabetes mellitus: 2 = Impaired glucose tolerance (in absence of intercurrent illness); 4 = Non-insulin-dependent diabetes (NIDDM) (tablets); 5 = Requiring insulin (irrespective of treatment at onset).
i. Cognition (Patients undergo testing using WTAR, Symbol Search and Speed of Comprehension Test): 2 = Combined centiles 30 – 59; 3 = Combined centiles 15 – 29; 4 = Combined centiles 5 – 14.
j. Seizures: 2 = Myoclonic or simple partial seizures only; 3 = Multiple absence, complex partial, or myoclonic seizures affecting function or single generalised seizure. All other patients with MELAS syndrome had an NMDAS score of 1 = Asymptomatic but past history.
k. Cerebellar ataxia: 2 = Reasonably steady gate. Unable to maintain heel-toe walking or mild upper limb (UL) dysmetria; 3 = Ataxic gait (but walks unaided) or UL intention tremor and past-pointing. Unable to walk heel-toe – falls immediately.
l. Respiratory muscle weakness: 2 = FVC < 75% predicted; 3 = FVC < 65% predicted.
m. Ptosis: 3 = Bilateral ptosis obscuring < 1/3 or unilateral ptosis obscuring > 1/3 of pupil or prior unilateral surgery.
n. Cardiac: 3 = Sustained or symptomatic arrhythmia, LVH or cardiomyopathy. Dilated chambers or reduced function on echo. Mobitz II AV block or greater; 4 = Requires pacemaker, defibrillator, arrhythmia ablation, or LVEF < 35% on echocardiogram.

Table S2 Summary demographics and clinical characteristics for patients with m.3243A>G-mitochondrial disease

	Sex	Age	Height (m)	Weight (kg)	BMI (kg/m^2)	Smoking	Work	Disease severity[c]	Heteroplasmy (%)[c]	SLE No.	Age[b]	Clinical characteristics	Medication
MELAS 01	M	55	1.63	65.6	25.0	Never	No	42.5<br>Mod	28, 98, 87	2	42	MELAS, encephalopathy, SNHL, impaired glucose tolerance, chronic constipation, LVH, cognitive impairment	Simvastatin 80mg OD,<br>Aspirin 75mg OD,<br>Levetiracetam 1000mg BD,<br>Citalopram 10mg OD,<br>Ramipril 10mg OD,<br>Bisoprolol 5mg OD
02	F	29	1.59	57	22.1	Never	No	33.3<br>Mod	21, 54, 42	1	28	MELAS, SNHL, migraine, chronic constipation, ataxia	Bisocodyl,<br>Carbimazole 50mg alt. d.<br>Folic acid,<br>Gabapentin 1200mg TDS,<br>Levetiracetam 1000/1250,<br>Progresterone only pills,<br>Omeprazole,<br>Clobazam 10mg (PRN),<br>CoQ10<br>L-arginine
03	M	50	1.66	58.5	22.7	Never	No	43.5<br>Mod	16, 65, 72	2	46	MELAS, SNHL, DM, ataxia myopathy, exercise intolerance, CKD, LVH, cognitive impairment	Insulin,<br>Atorvastatin,<br>Irbersartan
04	M	30	1.72	52	19.4	Never	No	53.7<br>Severe	39, 100, 95	2	24	MELAS, SNHL, DM, migraine, chronic constipation, cognitive impairment, ataxia, myopathy, exercise intolerance, respiratory muscle weakness, ptosis, psychiatric involvement	Gliclazide 80 mg OD,<br>Lamotrigine 200mg BD,<br>Clobazam 10mg OD,<br>Gabapentin 300mg BD,<br>Mirtazapine 30mg OD,<br>Accrete D3 1 tab BD,<br>Pyridoxine 10mg OD,<br>Rizatriptan,<br>Perampanel 6mg OD,<br>Escitalopram 10mg OD
05	F	30	1.59	41.8	17.4	NA	No	30<br>Mod	23, 59, 57	2	29	MELAS, SNHL, migraine, occasional GI symptoms, myopathy, exercise intolerance, respiratory muscle weakness, ptosis, cognitive impairment, psychiatric involvement	Perampanel,<br>Levetiracetam
06	F	28	1.49	47.3	19.8	NA	No	55.9<br>Severe	33, 81, 81	2	27	MELAS, encephalopathy, SNHL, migraine, chronic constipation, myopathy, exercise intolerance, respiratory muscle weakness, cognitive impairment, psychiatric involvement	Insulin (lantus, novorapiod)<br>Aspirin,<br>Bisoprolol,<br>Perindopril,<br>Simvastatin,<br>CoQ10,<br>Riboflavin

	Sex	Age	Height (m)	Weight (kg)	BMI (kg/m²)	Smoking	Work	Disease severity[c]	Heteropl-asmy (%)[c]	SLE No.	Age[b]	Clinical characteristics	Medication
Non-Melas 01	F	23	1.59	61.4	22.0	Never	Yes	26.9 Mod	44, 96, 65	0	NA	SNHL, DM, migraine, severe gut dysmotility, exercise intolerance, myopathy, psychiatric involvement	Insulin,<br>Bisoprolol 2.5mg OD,<br>CoQ10 100mg TDS,<br>Sertraline,<br>Docuastate ,Movicol
02	M	32	1.71	66.4	23.5	Never	Yes	3.1 Mild	28, 76, 90	0	NA	Asymptomatic – other than occasional GI symptoms	Nil
03	M	24	1.71	71	24.9	NA	Yes	4.1 Mild	24, 56, 80	0	NA	Asymptomatic – other than occasional GI symptoms	Creatine,<br>Nitrate supplement
04	M	35	1.82	92	28.5	Former	Yes	4.1 Mild	6, 18, 82	0	NA	Asymptomatic – other than mild hearing loss	Nil
05	M	43	1.68	79.3	27.6	NA	Yes	59.1 Severe	27, 96, 85	0	NA	SNHL, DM, migraine, occasional GI symptoms, exercise intolerance, myopathy, ataxia, WPW (had ablation), psychiatric involvement	Insulin (lantus, novorapiod)<br>Aspirin,<br>Bisoprolol,<br>Perindopril,<br>Simvastatin,<br>CoQ10<br>Riboflavin
06	F	38	1.60	93	30.9	Never	Yes	11.4 Mild	20, 62 56	0	NA	DM, chronic constipation	Insulin (novorapid, glargine),<br>Rampiril,<br>Trenaxemic acid
07	F	61	1.64	95.4	32.9	Former	No	36.5 Mod	NA, NA, 20	0	NA	DM, migraine, chronic constipation, exercise intolerance, ptosis, cognitive impairment, psychiatric involvement	Gabapentin,<br>Amlodipine,<br>Atorvastatin,<br>Co-dydramol,<br>Metformin,<br>Gliclazide,<br>Paracetamol,<br>Propanolol,<br>Trazadone,<br>Dihydrocodeine
08	F	37	1.62	60.6	24.4	Current	Yes	25.8 Mod	10, 31, 55	0	NA	Migraine, occasional GI symptoms, exercise intolerance, myopathy, ptosis	Amoxicillin for LRTI,<br>Cannabis (illicit)
09	M	51	1.75	51	26.1	Never	Yes	21.8 Mild	17, 70, 76	0	NA	SNHL, DM, migraine, occasional GI symptoms, psychiatric involvement	Fluoxetine 40mg OD,<br>Lansoprazole 30mg OD,<br>Metformin 1g BD,<br>Statin,<br>Alogliptin 6.25mg OD,<br>CoQ10 500mg OD,<br>Vitamin D,<br>Anti-histamine

	Sex	Age	Height (m)	Weight (kg)	BMI (kg/m^2)	Smoking	Work	Disease severity[c]	Heteropl-asmy (%)[c]	SLE No.	Age[b]	Clinical characteristics	Medication
10	M	50	1.76	57.9	18.6	Current	No	24.9<br>Mod	24 99, 85	0	NA	Migraine, chronic constipation, myopathy exercise intolerance, psychiatric involvement	Ensure plus,<br>CoQ10 100mg TDS,<br>Bezfaibrate 200mg TDS,<br>Laxido 1-2 sachets/day

Data are individual patient demographics and clinical characteristics for the m.3243A>G-mitochondrial disease patients.
Abbreviations: alt. d. = alternate days; BD = twice a day; CoQ10, coenzyme Q10; CKD = chronic kidney disease; DM, diabetes mellitus; GI = gastro-intestinal; LVH = left ventricular hypertrophy; MELAS = mitochondrial encephalomyopathy with lactic acidosis stroke-like episodes; NA, not available; NMDAS = Newcastle Mitochondrial Disease Scale for Adults; OD = once a day; PRN = as needed; SNHL = Sensorineural hearing loss; SLE = stroke-like episode/s; TDS = 3 times a day; WPW = Wolff Parkinson White syndrome.

a. Age of first stroke-like episode
b. Heteroplasmy levels for blood, age-adjusted blood and urine.
c. NMDAS. Total = scaled score. Higher scores indicate greater disease seventy.

Table S3 Correlation matrix between demographics, disease severity, self-reported fatigue and performance outcomes in MELAS syndrome

	1	2	3	4	5	6	7	8	9	10	11	12	13	14	15	16	17	18	19	20	21	22	23	24	25	26	27
**1.** Age (years)	1																										
**2.** Weight (kg)	0.78	1																									
**3.** BMI (kg/m^2)	0.59	**0.82***	1																								
**4.** Blood heteroplasmy (%)[a]	0.28	0.31	0.13	1																							
**5.** NMDAS (total)	-0.04	0.04	0.06	0.71	1																						
**6.** FIS (total)	-0.51	0.03	0.32	-0.51	0.01	1																					
**7.** Peak HR (bpm)	-0.27	0.1	0.27	0.83	0.73	0.61	1																				
**8.** Peak SV (L/min)	-0.81	-0.93	-0.93	-0.68	-0.35	0.25	-0.56	1																			
**9.** Peak SV index (L/min)	-0.81	**-0.98***	-0.91	-0.7	-0.52	0.07	-0.54	**0.97***	1																		
**10.** Peak QT (L/min)	-0.92	-0.67	-0.85	0.14	0.34	0.85	0.26	0.64	0.57	1																	
**11.** Peak cardiac index (L/min)	**-1#**	-0.88	**-0.97***	-0.13	-0.01	0.59	0.04	0.81	0.8	0.94	1																
**12.** Peak VO_2 (ml/kg/min)	**-0.9***	**-0.98#**	-0.78	-0.53	-0.01	0.46	-0.11	**0.99***	**0.98***	0.7	0.88	1															
**13.** AT (ml/kg/min)	-0.85	**-0.89***	-0.59	-0.5	0.16	0.67	-0.03	**1†**	**0.97***	0.65	0.81	**0.96#**	1														
**14.** Peak a-vO2 diff	-0.19	-0.47	-0.44	-0.9	-0.38	-0.05	-0.94	0.73	0.65	0.05	0.2	0.61	0.72	1													
**15.** Peak power (W/kg)	-0.68	-0.69	-0.38	-0.06	-0.17	0.24	0.36	0.33	0.52	0.35	0.57	0.56	0.48	-0.26	1												
**16.** TUG (sec)	-0.01	0.47	0.44	-0.46	-0.45	0.67	-0.36	-0.62	-0.75	-0.58	-0.8	-0.79	-0.77	0	**-0.92***	1											
**17.** Handgrip strength (kg)	0.65	**0.89***	0.81	0.7	0.58	0.11	0.44	-0.87	**-0.96***	-0.43	-0.71	-0.81	-0.66	-0.53	-0.62	0.63	1										
**18.** Inactivity	**0.87***	0.69	0.37	0.28	0.21	-0.51	-0.34	-0.54	-0.68	-0.58	-0.78	-0.73	-0.66	0.11	**-0.97#**	-0.02	0.68	1									
**19.** Light PA	0.1	0.01	0.45	-0.16	-0.36	0.01	0.15	-0.35	-0.14	-0.64	-0.41	-0.1	-0.05	-0.3	0.56	-0.04	-0.12	-0.37	1								
**20.** Moderate PA	-0.27	-0.13	0.41	-0.24	-0.18	0.52	0.39	-0.3	-0.07	-0.51	-0.28	0.16	0.27	-0.36	0.68	0.13	-0.13	-0.62	**0.87***	1							
**21.** Vigorous PA	-0.36	-0.34	0.16	0.16	0.51	0.32	0.6	-0.4	-0.19	-0.21	-0.09	0.37	0.55	-0.72	0.54	-0.4	0	-0.44	0.48	0.69	1						
**22.** MVPA	-0.28	-0.14	0.41	-0.23	-0.15	0.52	0.41	-0.3	-0.07	-0.51	-0.28	0.18	0.29	-0.37	0.68	0.11	-0.13	-0.62	**0.87***	**1**	0.72	1					
**23.** Inactivity-30min	0.5	0.41	-0.02	0.3	0.38	-0.34	-0.28	-0.12	-0.35	0.04	-0.23	-0.39	-0.36	0.26	**-0.93***	-0.05	0.51	**0.85***	-0.8	**-0.89***	-0.51	**-0.88***	1				
**24.** MVPA-5min	-0.43	0.04	0.34	-0.55	-0.41	**0.88***	0.39	-0.03	0.2	-0.31	-0.03	0.38	0.49	-0.22	0.74	0.79	-0.26	-0.56	0.36	0.65	0.14	0.63	-0.6	1			
**25.** MVPA-10min	0.62	0.76	0.76	0.36	-0.19	-0.2	0.29	-0.92	-0.8	-0.67	-0.72	-0.86	-0.85	-0.77	-0.07	0.27	0.58	0.24	0.54	0.31	-0.11	0.29	-0.17	0.15	1		
**26.** Steps (week)	0.05	0.13	0.56	-0.06	-0.27	0.19	0.35	-0.52	-0.3	-0.62	-0.45	-0.19	-0.12	-0.51	0.56	0.11	0.04	-0.41	**0.96#**	**0.92***	0.51	**0.91***	-0.81	0.5	0.63	1	
**27.** Sleep duration	-0.49	-0.07	-0.4	0.06	0.08	0.26	0.18	0.35	0.22	0.91	0.71	0.19	0.06	-0.1	-0.12	0.41	-0.03	-0.2	-0.72	-0.45	-0.42	-0.45	0.25	0.21	-0.31	-0.56	1

-1.0

1.0

Data are Pearsons's rho. Strength of association denoted by -1.0 (darkest pink) to 1.0 (darkest blue). Bold denotes significant values: * $P < 0.05$, # $P < 0.01$, † $P < 0.001$. Abbreviations: AT = anaerobic threshold; BMI = body mass index; FIS = Fatigue Impact Scale; MVPA = moderate vigorous physical activity; NMDAS = Newcastle Mitochondrial Disease Scale for Adults; PA = physical activity; QT = cardiac output; SV = stroke volume; TUG = Timed-Up and Go.

Table S4 Correlation matrix between demographics, disease severity, self-reported fatigue and performance outcomes in non-MELAS

	1	2	3	4	5	6	7	8	9	10	11	12	13	14	15	16	17	18	19	20	21	22	23	24	25	26	27
**1.** Age (years)	1																										
**2.** Weight (kg)	0.16	1																									
**3.** BMI (kg/m^2)	0.13	0.93†	1																								
**4.** Blood heteroplasmy (%)[a]	0.18	-0.39	-0.23	1																							
**5.** NMDAS (total)	0.47	0.01	0.12	0.52	1																						
**6.** FIS (total)	0.65	-0.13	-0.02	0.68	0.86#	1																					
**7.** Peak HR (bpm)	-0.69*	-0.12	-0.25	-0.59	-0.88#	-0.88#	1																				
**8.** Peak SV (L/min)	0.83#	0.31	0.27	0.36	0.67*	0.77*	-0.74*	1																			
**9.** Peak SV index (L/min)	0.82#	0.16	0.17	0.54	0.77#	0.85*	-0.83#	0.98†	1																		
**10.** Peak QT (L/min)	0.59	0.25	0.08	-0.11	0.01	0.19	-0.11	0.71*	0.57	1																	
**11.** Peak cardiac index (L/min)	0.6	-0.03	-0.14	0.1	0.06	0.26	-0.12	0.68*	0.61	0.94†	1																
**12.** Peak VO_2 (ml/kg/min)	-0.33	-0.39	-0.56	-0.19	-0.69*	-0.55	0.8#	-0.42	-0.45	0.14	0.26	1															
**13.** AT (ml/kg/min)	-0.49	-0.41	-0.52	-0.13	-0.63*	-0.51	0.78#	-0.43	-0.45	0.11	0.24	0.93†	1														
**14.** Peak a-vO2 diff	-0.56	-0.11	-0.27	-0.34	-0.75*	-0.72*	0.88#	-0.65*	-0.69*	-0.19	-0.16	0.87#	0.79#	1													
**15.** Peak power (W/kg)	-0.37	-0.24	-0.48	-0.3	-0.71*	-0.63	0.81#	-0.34	-0.42	0.32	0.35	0.92†	0.89#	0.77#	1												
**16.** TUG (sec)	0.71*	0.15	0.12	0.41	0.86#	0.82#	-0.85#	0.75*	0.82#	0.17	0.16	-0.54	-0.65*	-0.59	-0.57	1											
**17.** Handgrip strength (kg)	-0.49	0.18	-0.06	-0.57	-0.64*	-0.67*	0.71*	-0.36	-0.47	0.15	0.04	0.55	0.65*	0.63*	0.72*	-0.58	1										
**18.** Inactivity	0.91#	0.13	0.2	0.11	0.65	0.79*	-0.74*	0.75*	0.77*	0.37	0.38	-0.42	-0.57	-0.57	-0.54	0.82#	-0.69*	1									
**19.** Light PA	-0.59	0.15	0.02	-0.61	-0.43	-0.51	0.44	-0.5	-0.57	-0.26	-0.41	0.03	0.18	0.24	0.24	-0.5	0.75*	-0.7*	1								
**20.** Moderate PA	-0.59	0.01	-0.14	-0.9#	-0.76*	-0.92#	0.81#	-0.74*	-0.82#	-0.27	-0.36	0.41	0.37	0.58	0.48	-0.73*	0.71*	-0.68*	0.75*	1							
**21.** Vigorous PA	-0.32	-0.18	-0.26	0.09	-0.51	-0.55	0.64	-0.42	-0.43	-0.11	-0.01	0.75*	0.57	0.78*	0.58	-0.37	0.12	-0.27	-0.33	0.2	1						
**22.** MVPA	-0.62	-0.05	-0.2	-0.77*	-0.83#	-0.98†	0.92#	-0.79*	-0.87#	-0.28	-0.32	0.6	0.51	0.76*	0.61	-0.77*	0.67*	-0.69*	0.56	0.95†	0.49	1					
**23.** Inactivity-30min	0.77*	-0.16	-0.05	0.46	0.56	0.73*	-0.58	0.64	0.7*	0.34	0.46	-0.13	-0.26	-0.37	-0.33	0.66	-0.74*	0.89#	-0.91#	-0.79*	0.05	-0.69*	1				
**24.** MVPA-5min	-0.71*	-0.12	-0.22	-0.52	-0.33	-0.69	0.6	-0.76*	-0.75*	-0.69*	-0.71*	0.29	0.24	0.61	0.24	-0.34	0.42	-0.52	0.52	0.71*	0.33	0.74*	-0.57	1			
**25.** MVPA-10min	-0.34	-0.13	-0.24	0.04	-0.55	-0.59	0.67*	-0.43	-0.45	-0.1	-0.02	0.76*	0.59	0.82#	0.62	-0.38	0.19	-0.31	-0.26	0.24	1†	0.52	-0.01	0.36	1		
**26.** Steps (week)	-0.81#	-0.08	-0.24	-0.7	-0.66	-0.87#	0.82#	-0.8#	-0.85#	-0.41	-0.46	0.45	0.47	0.66	0.55	-0.7*	0.74*	-0.85#	0.76*	0.91#	0.3	0.91#	-0.87#	0.82#	0.35	1	
**27.** Sleep duration	0.01	-0.12	0.08	0.97†	0.39	0.47	-0.43	0.29	0.39	0.02	0.15	-0.22	-0.08	-0.34	-0.25	0.17	-0.48	0.04	-0.51	-0.72*	0.06	-0.62	0.31	-0.55	0.02	-0.5	1

Data are Pearsons's rho. Strength of association denoted by -1.0 (darkest red) to 1.0 (darkest blue). Bold denotes significant values: * $P < 0.05$, # $P < 0.01$, † $P < 0.001$. Abbreviations: AT = anaerobic threshold; BMI = body mass index; FIS = Fatigue Impact Scale; MVPA = moderate vigorous physical activity; NMDAS = Newcastle Mitochondrial Disease Scale for Adults; PA = physical activity; QT = cardiac output; SV = stroke volume; TUG = Timed-Up and Go.

Table S5 Significant correlation matrix between demographics, disease severity, self-reported fatigue and performance outcomes in MELAS syndrome

	1	2	3	4	5	6	7	8	9	10	11	12	13	14	15	16	17	18	19	20	21	22	23	24	25	26	27
**1.** Age (years)																											
**2.** Weight (kg)																											
**3.** BMI (kg/m^2)		0.82*																									
**4.** Blood heteroplasmy (%)[a]																											
**5.** NMDAS (total)																											
**6.** FIS (total)																											
**7.** Peak HR (bpm)																											
**8.** Peak SV (L/min)																											
**9.** Peak SV index (L/min)		-0.98*						0.97*																			
**10.** Peak QT (L/min)																											
**11.** Peak cardiac index (L/min)	-1#		-0.97*																								
**12.** Peak VO_2 (ml/kg/min)	-0.9*	-0.98#						0.99*	0.98*																		
**13.** AT (ml/kg/min)		-0.89*						1†	0.97*			0.96#															
**14.** Peak a-vO2 diff																											
**15.** Peak power (W/kg)																											
**16.** TUG (sec)															-0.92*												
**17.** Handgrip strength (kg)		0.89*							-0.96*																		
**18.** Inactivity	0.87*														-0.97#												
**19.** Light PA																											
**20.** Moderate PA																			0.87*								
**21.** Vigorous PA																											
**22.** MVPA																			0.87*								
**23.** Inactivity-30min															-0.93*			0.85*		-0.89*		-0.89*					
**24.** MVPA-5min						0.88*																					
**25.** MVPA-10min																											
**26.** Steps (week)																			0.96#	0.92*		0.91*					
**27.** Sleep duration																											

Data are Pearsons's rho. Strength of association denoted by -1.0 (darkest red) to 1.0 (darkest blue). Bold denotes significant values: * $P < 0.05$, # $P < 0.01$, † $P < 0.001$. Table displays only correlations that reached statistical significance. Abbreviations: AT = anaerobic threshold; BMI = body mass index; FIS = Fatigue Impact Scale; MVPA = moderate vigorous physical activity; NMDAS = Newcastle Mitochondrial Disease Scale for Adults; PA = physical activity; QT = cardiac output; SV = stroke volume; TUG = Timed-Up and Go. [a] Heteroplasmy levels for blood were age adjusted

Table S6 Significant correlation matrix between demographics, disease severity, self-reported fatigue and performance outcomes in non-MELAS

	1	2	3	4	5	6	7	8	9	10	11	12	13	14	15	16	17	18	19	20	21	22	23	24	25	26	27
**1.** Age (years)																											
**2.** Weight (kg)																											
**3.** BMI (kg/m²)		**0.93†**																									
**4.** Blood heteroplasmy (%)[a]																											
**5.** NMDAS (total)																											
**6.** FIS (total)					**0.86#**																						
**7.** Peak HR (bpm)	**-0.69***				**-0.88#**	**-0.88#**																					
**8.** Peak SV (L/min)	**0.83#**				**0.67***	**0.77***	**-0.74***																				
**9.** Peak SV index (L/min)	**0.82#**				**0.77#**	**0.85***	**-0.83#**	**0.98†**																			
**10.** Peak QT (L/min)								**0.71***																			
**11.** Peak cardiac index (L/min)								**0.68***		**0.94†**																	
**12.** Peak VO_2 (ml/kg/min)					**-0.69***		**0.8#**																				
**13.** AT (ml/kg/min)					**-0.63***		**0.78#**					**0.93†**															
**14.** Peak a-vO2 diff					**-0.75***	**-0.72***	**0.88#**	**-0.65***	**-0.69***			**0.87#**	**0.79#**														
**15.** Peak power (W/kg)					**-0.71***		**0.81#**					**0.92†**	**0.89#**	**0.77#**													
**16.** TUG (sec)	**0.71***				**0.86#**	**0.82#**	**-0.85#**	**0.75***	**0.82#**				**-0.65***														
**17.** Handgrip strength (kg)					**-0.64***	**-0.67***	**0.71***						**0.65***	**0.63***	**0.72***												
**18.** Inactivity	**0.91#**					**0.79***	**-0.74***	**0.75***	**0.77***							**0.82#**	**-0.69***										
**19.** Light PA																	**0.75***	**-0.7***									
**20.** Moderate PA				**-0.9#**	**-0.76***	**-0.92#**	**0.81#**	**-0.74***	**-0.82#**							**-0.73***	**0.71***	**-0.68***	**0.75***								
**21.** Vigorous PA												**0.75***		**0.78***													
**22.** MVPA				**-0.77***	**-0.83#**	**-0.98†**	**0.92#**	**-0.79***	**-0.87#**					**0.76***		**-0.77***	**0.67***	**-0.69***		**0.95†**							
**23.** Inactivity-30min	**0.77***					**0.73***			**0.7***								**-0.74***	**0.89#**	**-0.91#**	**-0.79***		**-0.69***					
**24.** MVPA-5min	**-0.71***							**-0.76***	**-0.75***	**-0.69***	**-0.71***									**0.71***		**0.74***					
**25.** MVPA-10min							**0.67***					**0.76***		**0.82#**							**1†**						
**26.** Steps (week)	**-0.81#**					**-0.87#**	**0.82#**	**-0.8#**	**-0.85#**							**-0.7***	**0.74***	**-0.85#**	**0.76***	**0.91#**		**0.91#**	**- 0.87#**	**0.82#**			
**27.** Sleep duration				**0.97†**																**-0.72***							

Data are Pearsons's rho. Strength of association denoted by -1.0 (darkest red) to 1.0 (darkest blue). Bold denotes significant values: * $P < 0.05$, # $P < 0.01$, † $P < 0.001$. Table displays only correlations that reached statistical significance. Abbreviations: AT = anaerobic threshold; BMI = body mass index; FIS = Fatigue Impact Scale; MVPA = moderate vigorous physical activity; NMDAS = Newcastle Mitochondrial Disease Scale for Adults; PA = physical activity; QT = cardiac output; SV = stroke volume; TUG = Timed-Up and Go. [a] Heteroplasmy levels for blood were age adjusted

Table S7 Correlation matrix between PROs–PerfOs (patient-reported and performance outcomes)

	**MELAS syndrome**									**Non-MELAS**								
	**Well-being**	**Health-related QOL – NMQ**			**Autonomic**	**Self-reported physical activity (IPAQ)**				**Well-being**	**Health-related QOL – NMQ**			**Autonomic**	**Self-reported physical activity (IPAQ)**			
	WEMWBS	Mobility	ADL	Energy/ Fatigue	COMPASS-31	Walking	Moderate	Vigorous	Total	WEMWBS	Mobility	ADL	Energy/ Fatigue	COMPASS-31	Walking	Moderate	Vigorous	Total
**1.** Age (years)	0.63	-0.54	0.14	**0.84***	-0.83	0.92	0.92	-0.6	0.91	**-0.76***	-0.65	-0.13	-0.61	0.45	-0.54	-0.14	0.13	-0.17
**2.** Weight (kg)	0.75	**-0.91***	-0.21	0.66	-0.46	0.91	0.93	-0.15	0.93	0.26	0.23	0.48	0.29	-0.28	-0.01	0.3	-0.3	-0.08
**3.** BMI (kg/m^2)	0.46	-0.76	-0.34	0.29	-0.4	0.68	0.73	0.23	0.73	0.14	0.12	0.4	0.16	-0.17	-0.17	0.01	-0.49	-0.31
**4.** Blood heteroplasmy (%)[a]	0.56	-0.46	0.6	0.31	-0.77	0.45	0.4	-0.72	0.41	-0.74	**-0.77***	**-0.74***	-0.67	0.64	**-0.72***	**-0.92#**	-0.5	**-0.78***
**5.** NMDAS (total)	-0.1	-0.07	0.12	-0.29	-0.31	0.01	-0.06	-0.8	-0.05	**-0.92#**	**-0.82#**	**-0.77***	**-0.69***	**0.85#**	-0.53	-0.5	-0.29	-0.48
**6.** FIS (total)	-0.45	-0.05	**-0.98#**	-0.68	0.84	-0.69	-0.64	0.99	-0.64	**-0.88#**	**-0.96†**	-0.61	**-0.92#**	**0.96†**	-0.38	-0.46	-0.08	-0.31
**7.** Peak HR (bpm)	0.24	-0.42	0.32	-0.28	0.7	0.06	0.05	0	0.06	**0.95#**	**0.89#**	0.61	**0.84#**	**-0.76***	0.62	0.48	0.33	0.53
**8.** Peak SV (L/min)	-0.93	**0.97***	-0.12	-0.94	1	-0.95	-0.94	0	-0.95	-0.65	**-0.67***	-0.32	-0.57	**0.67***	-0.43	-0.18	0.1	-0.16
**9.** Peak SV index (L/min)	-0.86	**0.97***	0.07	-0.87	1	-0.96	-0.96	0	-0.96	**-0.79***	**-0.79***	-0.47	**-0.68***	**0.76***	-0.54	-0.37	-0.02	-0.31
**10.** Peak QT (L/min)	-0.4	0.44	0.21	-0.84	1	-0.89	-0.9	0	-0.89	-0.1	-0.04	0.19	-0.04	0.13	0.05	0.35	**0.69***	0.46
**11.** Peak cardiac index (L/min)	-0.55	0.67	0.31	-0.91	1	-0.9	-0.91	0	-0.9	-0.23	-0.16	0.05	-0.13	0.24	-0.1	0.08	0.63	0.3
**12.** Peak VO_2 (ml/kg/min)	**-0.88***	**0.94***	-0.1	-0.84	0.89	-0.99	-0.99	0	-0.99	0.48	0.51	0.41	0.54	-0.43	0.37	0.2	0.41	0.41
**13.** AT (ml/kg/min)	**-0.95***	**0.9***	-0.29	**-0.94***	0.79	-0.95	-0.95	0	-0.95	0.59	0.52	0.34	0.54	-0.33	0.53	0.13	0.37	0.42
**14.** Peak a-vO2 diff	-0.93	0.8	-0.68	-0.56	1	-0.43	-0.42	0	-0.43	**0.76***	0.66	0.56	**0.72***	-0.62	0.43	0.25	0.09	0.27
**15.** Peak power (W/kg)	-0.24	0.41	0.65	-0.47	0.32	0.15	0.13	0	0.15	0.63	0.64	0.39	0.63	-0.49	0.6	0.5	0.61	**0.67***
**16.** TUG (sec)	0.19	-0.5	-0.77	0.04	0.65	-0.47	-0.41	**0.98***	-0.41	**-0.85***	**-0.83#**	-0.57	**-0.7***	**0.73***	-0.53	-0.29	-0.19	-0.37
**17.** Handgrip strength (kg)	0.58	-0.87	-0.25	0.39	-0.94	0.98	0.98	0	0.98	**0.89#**	**0.69***	0.48	**0.72***	-0.56	**0.78***	0.63	0.29	0.61
**18.** Inactivity	0.39	-0.39	-0.05	0.66	-0.83	0.59	0.56	-0.77	0.55	**-0.88***	**-0.77***	-0.21	-0.69	0.63	-0.59	-0.28	0.04	-0.27
**19.** Light PA	0.11	-0.08	0.18	0	-0.41	0.63	0.68	0.3	0.68	**0.92#**	0.52	0.21	0.43	-0.43	**0.81***	0.63	0.1	0.52
**20.** Moderate PA	-0.16	-0.01	-0.12	-0.42	0.02	0.2	0.26	0.69	0.26	**0.93#**	**0.94#**	0.56	**0.85#**	**-0.87#**	0.65	0.7	0.24	0.56
**21.** Vigorous PA	-0.44	0.25	0.05	-0.67	-0.15	0.45	0.47	0.23	0.48	**0.91***	0.52	0.38	0.59	-0.5	-0.14	-0.11	-0.04	-0.1
**22.** MVPA	-0.17	0	-0.11	-0.44	0.01	0.2	0.26	0.69	0.27	**0.95#**	**0.98†**	0.61	**0.92#**	**-0.92#**	0.52	0.57	0.19	0.45
**23.** Inactivity-30min	0.17	-0.18	-0.11	0.4	-0.2	0	-0.05	-0.74	-0.06	**-0.91***	**-0.73***	-0.24	-0.65	0.64	-0.65	-0.52	0.04	-0.36
**24.** MVPA-5min	-0.14	-0.19	-0.66	-0.42	0.72	-0.43	-0.37	**0.99***	-0.37	**0.84***	0.65	0.14	**0.72***	-0.54	0.45	0.34	-0.15	0.18
**25.** MVPA-10min	**0.87***	**-0.84***	0.21	0.66	-0.61	0.78	0.8	0	0.81	**0.92#**	0.55	0.41	0.62	-0.54	-0.08	-0.04	-0.02	-0.05
**26.** Steps (week)	0.2	-0.26	0.09	-0.04	-0.34	0.51	0.56	0.41	0.56	**0.95#**	**0.87#**	0.3	**0.83***	**-0.74***	0.64	0.55	0.11	0.44
**27.** Sleep duration	0.04	-0.12	-0.24	-0.17	0.69	-0.89	-0.9	0.33	-0.9	-0.51	-0.52	-0.58	-0.5	0.51	-0.64	**-0.89#**	-0.48	**-0.74***

Data are Pearsons's rho. Strength of association denoted by -1.0 (darkest red) to 1.0 (darkest blue). Bold denotes significant values: * $P < 0.05$, # $P < 0.01$, † $P < 0.001$. Abbreviations: ADL = activities of daily living; AT = anaerobic threshold; BMI = body mass index; MVPA = moderate-vigorous physical activity; NMDAS = Newcastle Mitochondrial Disease Scale for Adults; PA = physical activity; QT = cardiac output; SV = stroke volume; WEMWBS = Warwick-Edinburgh Mental Wellbeing Scale. [a] Heteroplasmy levels for blood were age adjusted

Table S8 Significant correlation matrix between PROs–PerfOs (patient-reported and performance outcomes)

	MELAS syndrome									Non-MELAS								
	Well-being	Health-related QOL – NMQ			Autonomic	Self-reported physical activity (IPAQ)				Well-being	Health-related QOL – NMQ			Autonomic	Self-reported physical activity (IPAQ)			
	WEMWBS	Mobility	ADL	Energy/ Fatigue	COMPASS-31	Walking	Moderate	Vigorous	Total	WEMWBS	Mobility	ADL	Energy/ Fatigue	COMPASS-31	Walking	Moderate	Vigorous	Total
**1.** Age (years)				**0.84***						**-0.76***								
**2.** Weight (kg)		**-0.91***																
**3.** BMI (kg/m²)																		
**4.** Blood heteroplasmy (%)[a]											**-0.77***	**-0.74***			**-0.72***	**-0.92#**		**-0.78***
**5.** NMDAS (total)										**-0.92#**	**-0.82#**	**-0.77***	**-0.69***	**0.85#**				
**6.** FIS (total)			**-0.98#**							**-0.88#**	**-0.96†**		**-0.92#**	**0.96†**				
**7.** Peak HR (bpm)										**0.95#**	**0.89#**		**0.84#**	**-0.76***				
**8.** Peak SV (L/min)		**0.97***									**-0.67***			**0.67***				
**9.** Peak SV index (L/min)		**0.97***								**-0.79***	**-0.79***		**-0.68***	**0.76***				
**10.** Peak QT (L/min)																	**0.69***	
**11.** Peak cardiac index (L/min)																		
**12.** Peak VO_2 (ml/kg/min)	**-0.88***	**0.94***																
**13.** AT (ml/kg/min)	**-0.95***	**0.9***		**-0.94***														
**14.** Peak a-vO2 diff										**0.76***			**0.72***					
**15.** Peak power (W/kg)																		**0.67***
**16.** TUG (sec)								**0.98***		**-0.85***	**-0.83#**		**-0.7***	**0.73***				
**17.** Handgrip strength (kg)										**0.89#**	**0.69***		**0.72***		**0.78***			
**18.** Inactivity										**-0.88***	**-0.77***							
**19.** Light PA										**0.92#**					**0.81***			
**20.** Moderate PA										**0.93#**	**0.94#**		**0.85#**	**-0.87#**				
**21.** Vigorous PA										**0.91***								
**22.** MVPA										**0.95#**	**0.98†**		**0.92#**	**-0.92#**				
**23.** Inactivity-30min										**-0.91***	**-0.73***							
**24.** MVPA-5min								**0.99***		**0.84***			**0.72***					
**25.** MVPA-10min	**0.87***	**-0.84***								**0.92#**								
**26.** Steps (week)										**0.95#**	**0.87#**		**0.83***	**-0.74***				
**27.** Sleep duration																**-0.89#**		**-0.74***

Data are Pearsons's rho. Strength of association denoted by -1.0 (darkest red) to 1.0 (darkest blue). Bold denotes significant values: * $P < 0.05$, # $P < 0.01$, † $P < 0.001$. Table displays only correlations that reached statistical significance. Abbreviations: ADL = activities of daily living; AT = anaerobic threshold; BMI = body mass index; MVPA = moderate-vigorous physical activity; NMDAS = Newcastle Mitochondrial Disease Scale for Adults; PA = physical activity; QT = cardiac output; SV = stroke volume; WEMWBS = Warwick-Edinburgh Mental Wellbeing Scale. [a] Heteroplasmy levels for blood were age adjusted
